# The CbrB Regulon: Promoter dissection reveals novel insights into the CbrAB expression network in *Pseudomonas putida*

**DOI:** 10.1371/journal.pone.0209191

**Published:** 2018-12-17

**Authors:** Rocío Barroso, Sofía M. García-Mauriño, Laura Tomás-Gallardo, Eloísa Andújar, Mónica Pérez-Alegre, Eduardo Santero, Inés Canosa

**Affiliations:** 1 Universidad Pablo de Olavide, Centro Andaluz de Biología del Desarrollo/ Consejo Superior de Investigaciones Científicas/ Junta de Andalucía, Seville, Spain; 2 Proteomics and Biochemistry unit, UPO/CABD/CSIC/JA, Seville, Spain; 3 Genomics unit, CABIMER/ CSIC/ Universidad de Sevilla/ Universidad Pablo de Olavide/ Junta de Andalucía, Seville, Spain; Universita degli Studi Roma Tre, ITALY

## Abstract

CbrAB is a high ranked global regulatory system exclusive of the Pseudomonads that responds to carbon limiting conditions. It has become necessary to define the particular regulon of CbrB and discriminate it from the downstream cascades through other regulatory components. We have performed *in vivo* binding analysis of CbrB in *P*. *putida* and determined that it directly controls the expression of at least 61 genes; 20% involved in regulatory functions, including the previously identified CrcZ and CrcY small regulatory RNAs. The remaining are porines or transporters (20%), metabolic enzymes (16%), activities related to protein translation (5%) and *orfs* of uncharacterised function (38%). Amongst the later, we have selected the operon *PP2810-13* to make an exhaustive analysis of the CbrB binding sequences, together with those of *crcZ* and *crcY*. We describe the implication of three independent non-palindromic subsites with a variable spacing in three different targets; CrcZ, CrcY and operon *PP2810-13* in the CbrAB activation. CbrB is a quite peculiar σ^N^—dependent activator since it is barely dependent on phosphorylation for transcriptional activation. With the depiction of the precise contacts of CbrB with the DNA, the analysis of the multimerisation status and its dependence on other factors such as RpoN o IHF, we propose a model of transcriptional activation.

## Introduction

The ability to recognize and convert external environmental stimuli into appropriate physiological responses is of fundamental importance for all organisms. Several distinct global regulatory networks can participate in co-ordinating gene expression programmes under different situations. In bacteria, signal transduction is predominantly mediated by two-component regulatory systems (TCSs) consisting of a sensor kinase (SK) and a cognate response regulator (RR), which are the principal tools by which bacteria sense and respond to different external signals [1].

Bacteria of the genus *Pseudomonas* are ubiquitous and very versatile, capable of adapting to very different habitats due to a complex regulatory network that ensures the appropriate responses to each context. The control on cellular carbon-nitrogen balance and determination of the order in which growth substrates are degraded is, in particular, mediated by the TCS CbrAB, which is exclusive of the *Pseudomonaceae* and found in the genera *Pseudomonas* [2–4] and *Azotobacter* [5]. The preferred carbon sources for the genus *Pseudomonas* are some organic acids or amino acids, rather than glucose, which is the preferred one in Enterobacteria. CbrAB TCS not only controls the assimilation of several amino acids (e.g. histidine, proline or arginine) as carbon or carbon and nitrogen sources but a number of other physiological functions, such as chemotaxis or stress tolerance [6]. CbrB directly activates the expression of the gene coding for the small regulatory RNA CrcZ *in P*. *putida*, *P*. *aeruginosa* and *P*. *syringae* [4, 7, 8], and in *P*. *putida* it also activates the expression of another regulatory RNA, CrcY [7, 9]. CrcZ and CrcY are directly related to the Carbon Catabolite Repression (CCR) in *P*. *putida* since they sequester and inactivate the protein responsible for such event; Crc, which acts in turn, as a translational repressor of its own targets [8–10]. Most Crc molecules can be sequestered by these sRNAs, although enough free Hfq is still available to perform other functions without the help of Crc. Maximal CCR is observed in the rich LB medium where the expression of CrcZ and CrcY is the lowest, and CCR is minimal in non-preferential carbon sources such as histidine or oxaloacetate [11].

Transcriptomic analyses in *Pseudomonas* indicate that CbrAB is likely to be an important transcriptional control system implicated in environmental sensing and adaptation [6, 12]. Indeed, the importance of CbrB is also reflected in its conservation among the *Pseudomonadaceae*, and also shows some specialization among some members which have evolved to regulate regulatory RNAs; CrcY in *P*. *aeruginosa* strains PAO1, PA14 or PA7, or in *P*. *mendocina* ymp., CrcZ and CrcY for *P*. *putida* KT2442, F1, GB-1 and W619 [9] or CrcZ, CrcY and CrcX in the case of *P*. *syringae* [13]. Interestingly, in *Azotobacter vinelandii* where the CbrAB system has been described, there also are orthologues of CrcZ and CrcY regulatory RNAs [4, 14]. The *Azotobacter* case is consistent with suggestions that *A*. *vinelandii* may in fact be a Pseudomonad based on genome similarity [15].

σ^N^-dependent transcriptional activators are usually dimers that bind to palindromic sequences located far upstream of the promoter. These regulators activate RNA-polymerase transcription at a distance *via* formation of a loop in the intervening DNA, which may be assisted by the protein IHF which binds between the promoter and the enhancer and induces a bend in the DNA upon binding that favours the interaction of the RNA polymerase-promoter closed complex and the enhancer-bound transcriptional activator [16, 17]. Tandem duplication of the palindromic sequences is very frequent to facilitate hexamerisation of the activator upon binding, an oligomerisation required for gaining ATP-hydrolysis and transcription activation capability [18]. However, CbrB has an unusual way of binding since it apparently binds two to three non-palindromic sequences spaced by 12–16 nucleotides [7, 19]. By combining mutational analyses and CbrB binding assays, this work aims to determine the sequences required for binding at selected promoters, the regulon directly controlled by CbrB and a pattern to find other regulated targets from sequence homology.

## Results

### *In vivo* CbrB binding analysis

Although our previous work using standard molecular methods such as Electrophoretic Mobility Shift Assays (EMSA) and DNA footprinting analysis showed that CbrB binds to regions upstream *crcZ* and *crcY*, and identified the precise binding contacts of CbrB in these promoter regions [7], we performed a genome-wide screen for CbrB binding sequences throughout the complete genome in order to better define the CbrB binding site and its primary regulon. For that purpose we performed a ChIP-Seq analysis where inmunoprecipitation of complexes of CbrB bound to its DNA targets were performed in the conditions of maximal induction of the *Cbr* system (i.e. OAA 20 mM as carbon source) [11]. After normalization and Peak calling filtration, a total of 314 inmunoprecipitated DNA fragments showed an enrichment of at least 2-fold with a p-value < 10^−10^. Out of those, 67 peaks contained at least one intergenic region, and some of them contained more than one. The resulting fragments were used for the search of putative σ^N^-dependent sequences in the right orientation (facing an *orf*) and a total of 61 individual DNA fragments following these premises were selected. To ensure that the putative CbrB binding sites were contained in these fragments, 100 bp were added upstream and downstream for the determination of a consensus sequence. The complete list of the candidate CbrB regulated genes is presented in Table 1, and some captures of the genomic regions that were further analysed are shown in S1 Fig. As an internal quality control for experiment, we detected a 4.3 and 2.6 -fold enrichment after inmunoprecipitation of the two previously characterised CbrB targets CrcZ and CrcY, respectively.

**Table 1:** List of the 61 selected targets after data mining in the ChIP-Seq analysis. The ID, annotation of the gene downstream the selected promoter region, fold enrichment of the peak, functional category and selected DNA fragments and peak coordinates in the *P*. *putida* KT2440 reference genome (GCF_000007565.1_ASM756v1) are shown. In bold, those genes whose expression was validated by RT-q PCR.

	ID	Annotation	Enrichment	Categories	DNA Fragment Coordinates		Peak Coordinates	
1	PP0009	*rpmH* 50S ribosomal protein L34	2.33	Translation	8847	9524	8860	9424
2	PP0036	transcriptional regulator	2.74	Regulatory	38954	39371	38757	39399
3	PP0153	hypothetical protein	4.32	hypothetical	161866	162366	161930	162266
4	PP0167	*paxB* toxin secretion ATP-binding protein	3.06	transport	193553	194537	193652	194437
5	PP0168	putative surface adhesion protein	3.06	transport	193553	194537	193652	194437
6	PP0397	*yeaG* protein kinase	2.53	Regulatory	484338	484816	483915	484716
7	PP0640	hypothetical protein	3.08	hypothetical	750762	751127	748555	751027
8	PP0731	pcs phosphatidylcholine synthase	4.19	enzymatic	850001	850910	850100	850810
9	PP0876	AraC family transcriptional regulator	2.93	Regulatory	1017151	1018026	1017172	1017926
**10**	**PP0952**	*rpoN* RNA polymerase sigma-54 factor	2.70	Regulatory	1095959	1096333	1095946	1096355
11	PP1116	resolvase family site-specific recombinase	2.09	enzymatic	1277917	1278833	1278016	1278868
12	PP1117	hypothetical protein	2.13	hypothetical	1279998	1281314	1280097	1281388
**13**	**PP1206**	*oprD* basic amino acid specific porin OprD	3.45	transport	1386355	1387119	1386334	1387019
14	PP1288	algD GDP-mannose 6-dehydrogenase	2.30	enzymatic	1474119	1474995	1474152	1474895
15	PP1691	hypothetical protein	3.03	hypothetical	1883564	1884708	1883621	1884716
16	PP1796	*aprDB* (pseudogene)	2.44	transport	2016394	2017010	2016493	2016929
17	PP1795	hypothetical protein	2.44	hypothetical	2016394	2017009	2016493	2016929
18	PP1947	hypothetical protein	2.32	hypothetical	2202041	2202420	2202121	2202416
19	PP2062	hypothetical protein	3.79	hypothetical	2345245	2346642	2344373	2346596
20	PP2063	hypothetical protein	3.79	hypothetical	2345245	2346643	2344373	2346596
21	PP2265	*folD*-II bifunctional methylenetetrahydro- folate dehydrogenase/ cyclohydrolase	3.38	enzymatic	2585408	2585860	2585507	2586482
22	PPt50	tRNA	3.38	Translation	2585408	2585860	2585507	2586482
23	PP2509	hypothetical protein	3.14	hypothetical	2856629	2858405	2856721	2858305
24	PP2541	transcriptional factor-like protein	3.38	Regulatory	2885949	2887273	2886048	2887173
**25**	**PP2810**	hypothetical protein	2.98	hypothetical	3202675	3203315	3202705	3203215
26	PP2858	hypothetical protein	2.75	hypothetical	3261156	3261619	3261202	3261519
27	PP3009	hypothetical protein	2.61	hypothetical	3401602	3401909	3401381	3403370
28	PP3012	hypothetical protein	2.61	hypothetical	3402885	3403137	3401381	3403370
29	PP3026	tail length determination protein	2.49	enzymatic	3412722	3413087	3412295	3412987
**30**	**PP3074**	*bhbP* D-beta-hydroxybutyrate permease	7.52	transport	3458628	3459012	3458727	3459050
31	PP3110	hypothetical protein	3.08	hypothetical	3519365	3521861	3519464	3521761
32	PP3238	transcriptional regulator PyrR	5.68	Regulatory	3674453	3674883	3674528	3674923
**33**	**PP3420**	sensor histidine kinase	3.38	Regulatory	3872278	3872784	3872243	3872733
34	PP3441	hypothetical protein	3.06	hypothetical	3899036	3899523	3899048	3899562
35	PP3540b	*crcY* regulatory RNA	2.57	Regulatory	4013035	4013244	4013134	4013436
**36**	**PP3656**	aromatic compound-specific porin	2.62	transport	4153120	4153479	4152926	4153689
37	PP3791	site-specific recombinase	2.40	enzymatic	4320663	4321941	4320709	4321841
38	PP3929	hypothetical protein	2.52	hypothetical	4433801	4434112	4433900	4434166
39	PP4035	*pydP* NCS1 family transporter PydP	4,28	transport	4547243	4547785	4547294	4547771
**40**	**PP4050**	*glgA* glycogen synthase	2,56	enzymatic	4563758	4564712	4320709	4321841
41	PP4070	hypothetical protein	3,42	hypothetical	4594982	4595629	4595080	4595540
42	PP4095	hypothetical protein	3,06	hypothetical	4630123	4631174	4629477	4631074
43	PP4283	GntR family transcriptional regulator	2,52	Regulatory	4874432	4874908	4874476	4874808
44	PP4309	NCS1 family transporter	3,29	transport	4899939	4900526	4899956	4900426
**45**	**PP4391**	*flgB* flagellar basal-body rod protein FlgB	2,44	motility	4983449	4983813	4983435	4983713
46	PP4424	HTH-type transcriptional regulator	2,36	Regulatory	5020010	5020411	5019442	5020946
47	PP4451	hypothetical protein	2,12	hypothetical	5049257	5050611	5048284	5050511
48	PP4471	*mgtE* magnesium transporter	2,26	transport	5078705	5079252	5078804	5080022
49	PP4474	*alaS* alanine—tRNA ligase	2,60	enzymatic	5084164	5084605	5084244	5084505
**50**	**PP4486**	*argT* lysine /arginine /ornithine ABC transporter substrate-binding protein	2,48	transport	5096664	5097230	5096657	5097130
51	PP4520	hypothetical protein	4,01	transport	5132506	5133016	5132454	5133090
**52**	**PP4643**	xanthine/uracil permease family protein	2,70	transport	5267050	5267512	5267006	5267505
**53**	***crcZ***	CrcZ regulatory RNA	4,32	Regulatory	5338050	5338276	5338149	5338829
54	PP_t72	tRNA	2,87	Translation	5361078	5361701	5360562	5361611
55	PP4739	hypothetical protein	2,40	hypothetical	5391562	5392963	5391661	5392863
56	PP4925	hypothetical protein	3,05	hypothetical	5604183	5604622	5602819	5604793
57	PP5129	hypothetical protein	3,46	hypothetical	5852185	5852693	5851137	5852593
58	PP5184	*spuI* glutamylpolyamine synthetase	2,88	transport	5912049	5912822	5912040	5912970
59	PP5212	iron-sulfur-binding oxidoreductase	2,66	transport	5944133	5944664	5944232	5944589
60	PP5376	hypothetical protein	2,64	hypothetical	6127775	6128896	6127848	6128801
61	PP5375	LysR family transcriptional regulator	2,64	Regulatory	6127775	6128895	6127848	6128801

Out of the selected DNA fragments, 12 of the *orfs* coded for proteins fitting the category of transport of different elements or molecules, 12 of them for regulatory proteins, thus possibly widening the CbrB regulatory cascade, 10 corresponded to proteins with putative enzymatic activities, one encoded FlgB involved in flagellar assembly with a putative role in motility, and 3 encoded proteins somehow related to translation. Finally, 23 of them corresponded to genes of unknown function annotated as hypothetical.

To determine if the DNA fragments selected in the *in vivo* binding experiment are associated with CbrB-dependent regulation, the expression of 12 targets including *crcZ* was analysed by quantitative RT-PCR (or β-galactosidase activity for PP2810) in a wild type and Δ*cbrB* backgrounds in a medium containing oxaloacetate as carbon source (Table 2). These targets were PP0952 encoding the sigma factor RpoN, PP1206 encoding the porin OprD, PP2810 and PP3009 encoding proteins of unassigned function, PP3074 encoding a putative permease, PP3420 encoding a putative histidine kinase, PP3656 encoding an aromatic compound-specific porin, PP4050 (*glgA*) encoding a glycogen synthase, PP4391 encoding the flagellar basal-body rod protein FlgB, PP4486 encoding a putative lysine/arginine/ornithine ABC transporter substrate-binding protein, PP4643 encoding a putative xanthine/uracil permease family protein and the gene *crcZ* encoding for the regulatory RNA CrcZ. Validation of the expression of the genes performed by quantitative RT-PCR showed that 7 of these targets were upregulated in the wild type strain (CbrB activated) and 3 of them were downregulated (CbrB repressed) (Table 2). Two of the genes (PP3009 and PP3656) did not show a significant difference in expression between the two strains by RT-qPCR analysis, although the fold enrichment in the ChIP-Seq was over the selected threshold.

**Table 2:** Validation of the ChIP-Seq data by RT-qPCR. Expression analysis of the selected genes was performed in a wild type and *cbrB* mutant strains collected at mid-exponential phase in a minimal medium containing OAA as carbon source. ChIP-Seq enrichment and mRNA levels determined by real-time RT-PCR are shown. Fold change values were calculated dividing the mRNA levels observed in the strain KT2442 by the levels in the *cbrB* mutant strain (MPO401). A minimum of three biological replicates for each sample in triplicates were performed. Stars designate p-values for the Student's t-test for unpaired samples not assuming equal variance and are referred to the wild type strain. *: p<0.05; **: p<0.01; ***:p<0.005.

ID	Gene-Annotation	ChIP-Seq enrichment	RT-qPCR Fold change	mRNA levels (arbitrary units)		t-test	
				KT2442	MPO401		
PP0952	*rpoN*- RNA polymerase sigma-54 factor	2.7	0.1	0.24 ± 0.06	1.78 ± 0.64	1,92E-03	**
PP1206	*oprD*- basic amino acid specific porin OprD	3.45	0.3	1.71 ± 0.85	5.77 ± 1.64	9,25E-04	***
PP2810	hypothetical protein	2.98	20.6^#^				
PP3009	hypothetical protein	2.61	1.0	0.30 ± 0.09	0.30 ± 0.11	9,74E-01	no differences
PP3074	*bhbP*- D-beta-hydroxybutyrate permease	7.52	5.7	0.68 ± 0.25	0.12 ± 0.09	7,94E-05	***
PP3420	sensor histidine kinase	3.38	11,7	0.70 ± 0.16	0.06 ± 0.02	8,53E-08	***
PP3656	aromatic compound-specific porin	2.62	1.1	0.015 ± 0.006	0.014 ± 0.003	6,20E-01	no differences
PP4050	*glgA*- glycogen synthase	2.56	0.7	0.23 ± 0.07	0.33 ± 0.04	9,85E-05	***
PP4391	*flgB-* flagellar basal-body rod protein FlgB	2.44	4.7	5.87 ± 0.92	1.26 ± 0.17	1,11E-09	***
PP4486	*argT-* lys/arg/orn ABC transp. substrate-binding protein	2.48	2.8	0.36 ± 0.11	0.13 ± 0.04	2,74E-03	**
PP4643	xanthine/uracil permease family protein	2.7	3.4	0.034 ± 0.01	0.01 ± 0.002	2,31E-03	**
crcZ	CrcZ- regulatory RNA	4.32	43.5	170.39 ± 39.17	3.92 ± 0.52	3,40E-03	**

^#^ Expression ratio for PP2810 was estimated as the β-galactosidase activity ratio using a transcriptional fusion to *lacZ* (see Fig 2).

**Fig 1:**
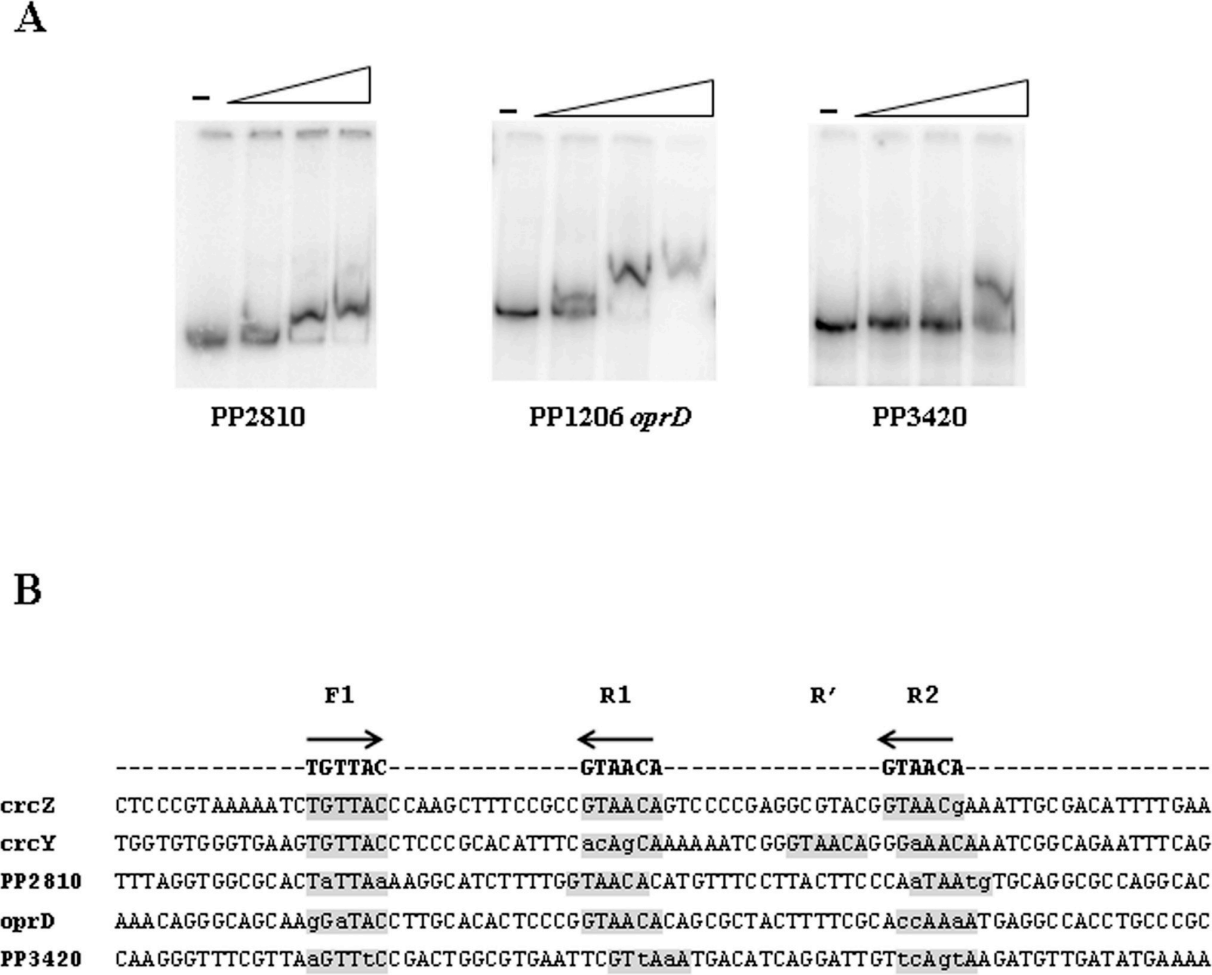
**(A)** Electrophoretic mobility shift assay (EMSA) for CbrB binding on PP2810, PP1206 (*oprD*) and PP3420 promoter regions. Band shift observed in linear dsDNA fragments containing the promoter regions in the presence of increasing amounts of CbrB (0, 1, 1.5 and 2 μM for PP2810 and 0, 0.5, 1 and 2 μM of CbrB for PP1206 and PP3420). A representative assay of three replicas performed is shown. **(B)** Sequence alignment of the promoter sequences of *crcZ*, *crcY*, *PP2810*, *oprD* and *PP3420* containing the CbrB binding sites. Grey boxes denote putative CbrB binding subsites in direct (type F) or reverse orientation (type R). In lowercase letters those bases that differ with the consensus sequence, which is denoted at the top.

Binding of CbrB to the promoter regions of some of the validated targets was analysed in order to acknowledge its direct interaction *in vitro*. We were able to detect a mobility shift for DNA fragments containing the promoter regions of PP2810, PP1206 and PP3420 (Fig 1A). However, a change in the mobility for PP4050 *glgA*, PP3074 permease or PP4391 (*flgB*) was not detected *in vitro* (S2 Fig). A possible reason could be that the affinity of CbrB itself for these targets is too low or other elements that are absent in the EMSA assay might be needed for CbrB binding.

Sequence inspection of the promoter region of the selected genes that were experimentally validated by EMSA for CbrB binding revealed a disposition coherent to that previously described for CrcZ and CrcY, that is, one subsite in the forward orientation (F1) and two additional subsites in the reverse orientation (R1 and R2) with a proper spacing between them (20–25 pb from the centres of two contiguous sites) (Fig 1B). All these binding subsites are topologically aligned at the same face of the DNA helix and those activated ones, such as in CrcZ and CrcY, at an appropriate distance from the σ^N^ promoter. In the *crcY* sequence an additional sequence denoted R’ is also found (Fig 1B). The sequence worst matching this arrangement was that of PP3420, in which a proper R2 subsite could not be recognised. In agreement with this, it actually was the sequence showing lowest affinity for CbrB (Fig 1A).

### Characterisation of the CbrB-mediated regulation of the *PP2810-13* target

In order to define a more precise CbrB consensus sequence we intensively studied the contribution of the putative CbrB binding subsites in the two previously characterised targets *crcZ* and *crcY*, coding for sRNAs, and in a new protein-coding operon target, comprising genes *PP2810* to *PP2813* [20], whose promoter region showed good binding of CbrB. The first two *orfs* in this operon do not show sequence homology to any characterised protein and are annotated as hypothetical proteins. Nevertheless *PP2812* is annotated as a transporter and *PP2813* as a BNR (bacterial neuraminidase repeat) domain-containing protein. Although the degree of sequence homology of *PP2810-13* in the databases was relatively low, structural modelling using the appropriate tools for protein homology detection and structure prediction (HHPred) [21] suggests they may conform a RND efflux pump. RND-type efflux pumps form multi-protein structures that bridge both the inner and outer membrane to expel diverse toxic compounds directly from the cell. Therefore, we decided to characterise its putative CbrB-mediated regulation.

**Fig 2:**
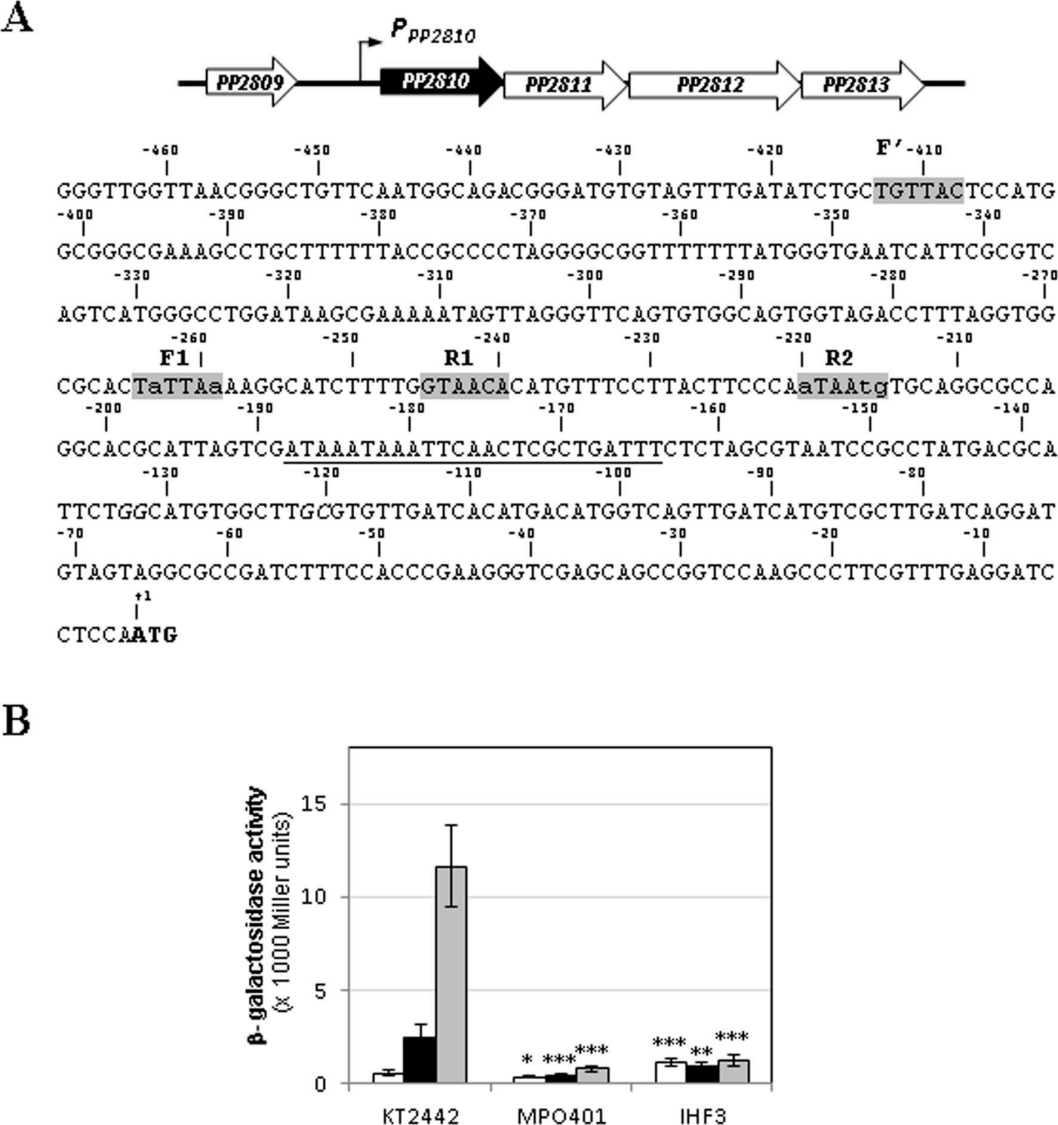
**(A)** Genomic context and sequence of operon *PP2810-PP2813*. Genomic context of PP2810 scheme in *Pseudomonas putida* along with its promoter sequence from the position -467 with respect to the initiation of translation (ATG, in bold). Sequences in grey correspond to CbrB binding subsites; the underlined sequence represents the **σ**^N^-dependent promoter sequence (GG-N_10_-GC) and the boxed sequence represents the putative IHF binding site. **(B)** *In vivo* expression from the *P*. *putida PP2810* promoter. Expression was measured as β-galactosidase activity of the *PP2810*::*lacZ* transcriptional fusions (plasmid pMPO420) of cultures of the KT2442 wild-type, the MPO401 (Δ*cbrB*) and IHF3 mutant strains, grown in LB (white bars) or in minimal medium containing succinate (black) or oxaloacetate (grey bars) as carbon sources. The values are the average of at least three independent assays. The error bars indicate the standard deviation of the means. Stars designate p-values for the Student's t-test for unpaired samples not assuming equal variance and are referred to the wild type strain. *: p<0.05; **: p<0.01; ***:p<0.005.

The intergenic region between the stop codon of *PP2809* and the ATG of *PP2810* comprises 472 bp and apparently contains all elements required for CbrB-activated transcription of the operon [7] (Fig 2A). In the first place, the consensus GG-N_10_-GC sequence characteristic of σ^N^-dependent promoters was found centred at -127 with respect to the start codon. Also, an IHF consensus sequence was detected in the DNA region comprising coordinates –165 to –189, in the region in between the putative CbrB and the σ^N^ binding sites. As previously described, IHF contributes to the correct assembly of transcription initiation complexes at a number of σ^N^-dependent promoters by inducing a curvature in the DNA, and assists CbrB-mediated transcription activation of *crcZ* and *crcY* (7). In addition to the three subsites putatively conforming a CbrB binding site, a sequence with perfect match with the forward subsite of the previously defined CbrB binding site (TGTTAC) [19] was found in the promoter region of the operon, although very far upstream, centred at -411, and separated from F1 by 149 nucleotides (denoted F’ in Fig 2A).

To validate the CbrB-mediated regulation of this operon and analyse its dependence on the above elements, a transcriptional fusion of the promoter region of PP2810 (containing 462 bp upstream of the ATG) to *lacZ* was constructed (plasmid pMPO420). Its expression was analysed by β-galactosidase activity in LB as a rich medium, in a minimal medium containing succinate, which constitute a preferential carbon source in *Pseudomonas*, or oxaloacetate as a non-preferential carbon source, in the wild type strain, in a *cbrB* mutant (strain MPO401) and in an *ihf* mutant (IHF3). β-galactosidase activity of the wild type strain bearing the plasmid pMPO420 showed basal expression levels that were not dependent on CbrB when grown in LB. However, it showed a 4.3- and 20.6-fold increase in a minimal medium containing succinate and oxaloacetate as carbon source, respectively, compared to LB (Fig 2B). Also, the activity in the Δ*cbrB* and *ihf* backgrounds was drastically reduced in the three media to basal levels, thus showing a clear dependence on both CbrB and IHF for activation of the operon transcription, as previously shown for *crcZ* and *crcY* [7].

### Mutational analysis of *crcZ*, *crcY* and *PP2810* promoter regions and definition of a CbrB binding site

We have previously shown a direct activation by CbrB and its direct binding to the promoter targets *crcZ*, *crcY* [7]. To more precisely define the CbrB contacts with the DNA, we performed mutation analyses of each potential subsite found at each promoter. The *crcZ*, *crcY* and *PP2810* targets contain at least three putative CbrB binding subsites (F1, R1 and R2). However, *crcY* presented an extra perfect R sequence (R’: GTAACA) centred at position -112 from the start codon (Fig 1B) and *PP2810* an extra perfect F sequence (F’: TGTTAC) centred at position -411 from the ATG (Fig 2A) with no counterparts in the other promoter regions. Deletion of the furthermost F’ subsite of *PP2810* (plasmid pMPO422) did not alter at all the ability of CbrB to activate PP2810 expression (12700 M.U. for pMPO420 in OAA versus 12300 for pMPO422), thus indicating that this putative subsite is dispensable. Contribution of the extra R’ subsite to the *crcY* transcriptional activation has been evaluated together with the other three CbrB binding subsites (F1, R1 and R2) by substitution mutagenesis (see below).

**Fig 3:**
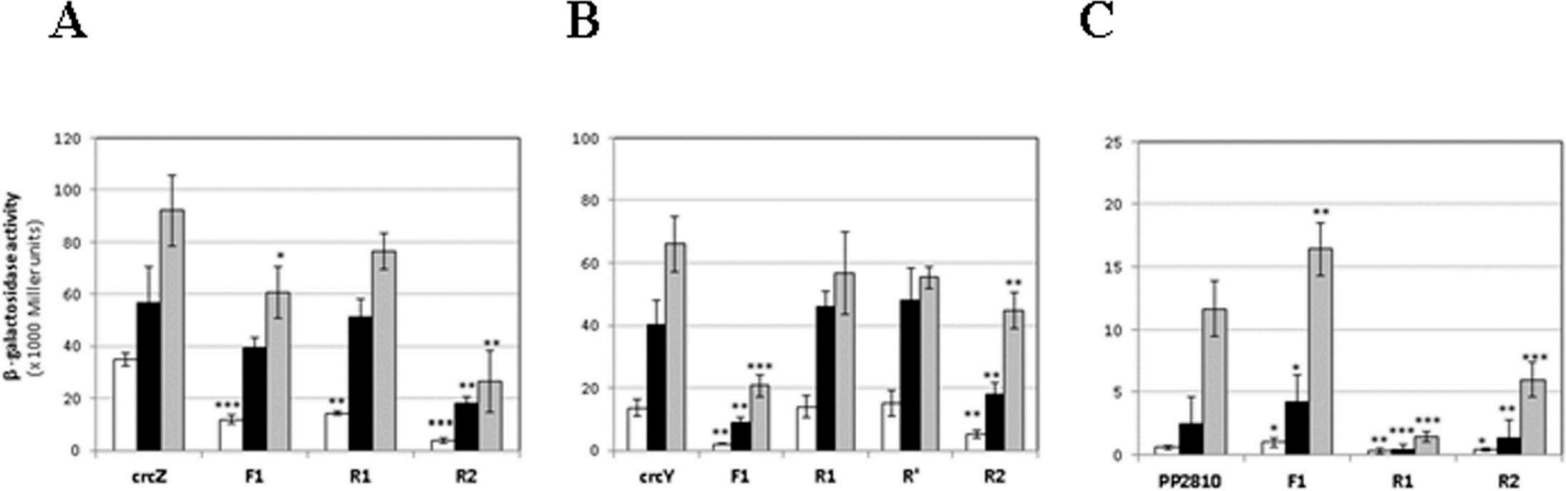
*In vivo* expression of *crcZ*, *crcY* and *PP2810* of *P*. *putida* and point mutagenesis of the CbrB binding sites. Expression was measured as **β**- galactosidase activity of the *crcZ* (A), *crcY* (B) and *PP2810* (C) *-lacZ* transcriptional fusions of the promoter region or the corresponding substituted sites F1, R1 or R2 in LB (white bars), minimal medium with succinate (black bars) and oxaloacetate (grey bars) as carbon source. Plasmids pMPO1316, pMPO1314 and pMPO422 contained the wild type sequences for *crcZ*, *crcY* and *PP2810*, respectively. Substitutions of F1, R1 (R’) and R2 were generated in plasmids pMPO436, pMPO437 and pMPO438 for *crcZ*, pMPO439, pMPO440 (pMPO441) and pMPO442 for *crcY* and plasmids pMPO425, pMPO426 and pMPO428 for *PP2810*. The values are the average of at least three independent assays. The error bars indicate the standard deviation of the means of at least three independent assays. Stars designate p-values for the Student's t-test for unpaired samples not assuming equal variance and are referred to the wild type sequence of each promoter. *: p<0.05; **: p<0.01; ***:p<0.005.

To determine the role of each subsite in transcription activation at the *crcZ*, *crcY* and *PP2810* promoter regions, 6-bp substitutions on F1, R1, R’ and R2 subsites were performed, and cloned into plasmid pMPO234, thus yielding transcriptional fusions of each promoter variant to *lacZ* (see legend of Fig 3 and S1 Table for sequence substitutions). β-galactosidase activity of the plasmids containing the wild type sequences and the substitution of each subsite for P_*crcZ*_, P_*crcY*_ and P_*PP2810*_ was assayed in LB and in minimal medium with succinate or oxaloacetate as the sole carbon sources (Fig 3).

The three targets were differentially expressed in the assayed media, and the induction was inversely proportional to the carbon availability in the medium; that is, maximal expression took place in the poorest medium (OAA), and lowest expression in the richest one (LB). The expression pattern for PP2810 coincided to that of *crcZ* and *crcY* previously described [7, 11], and was mainly dependent on CbrB as shown in a *cbrB* background where it was non-significant (Fig 2 and S3 Fig). The expression levels were highest for *crcZ* in OAA (92,200 M.U.) followed by those of *crcY* (66,000 M.U.) and finally those of *PP2810*, which showed almost 8-fold less expression than *crcZ*. However, the induction rate was maximal for PP2810, which was upregulated 20.6-fold in OAA relative to LB and 4.8-fold relative to succinate, as compared to the 2.6- and 1.6-fold induction rates, respectively, found for *crcZ*, or the 4.8 and 1.6 fold, respectively, found for *crcY* (Fig 3). This fact is mainly due to the low expression levels of *PP2810* in LB, which is in contrast with the low but significant CbrB-mediated activation observed for *crcZ* and *crcY* in this rich medium (S3 Fig) [7]. These values suggest that the PP2810 promoter region is the least sensitive to CbrB activity and requires the most carbon-limited medium for high levels of expression, while the CrcZ and CrcY small RNAs are highly activated in the presence of low amounts of active CbrB. Nevertheless, the three of them presented the same defined hierarchy of induction in response to the appropriate carbon source.

Substitution of each one of the subsites independently for each target affected the transcriptional activation in a different manner. Although all substitutions had a substantial effect on *crcZ* expression (Fig 3A) it was the substitution of R2 (GTAACg to taccgt) the one showing the most drastic effect for *crcZ* transcription (9.7-, 3.1- and 3.5- fold decrease in LB, Scc and OAA, respectively). In contrast, it was subsite F1 (TGTTAC to gactct) the subsite that most affected *crcY* expression (6.6-, 4.5- and 3.2-fold decrease in LB, Scc and OAA, respectively), whilst it was R1 (GTAACA to agcctc) the most important for *PP2810* expression (2.0-, 6.2- and 8.2-fold decrease in LB, Scc and OAA, respectively). Substitution of subsites R1 or R’ of P_*crcY*_ had no effect on *crcY* expression, and R2 had a milder effect than F1 (Fig 3B). Substitution of R2 in *PP2810* had an obvious but less severe effect than R1 substitution, whilst substitution of F1 even had a positive effect on *PP2810* expression (Fig 3C).

### CbrB binding affinities are consistent with the contribution of each of its binding subsites to expression

**Fig 4:**
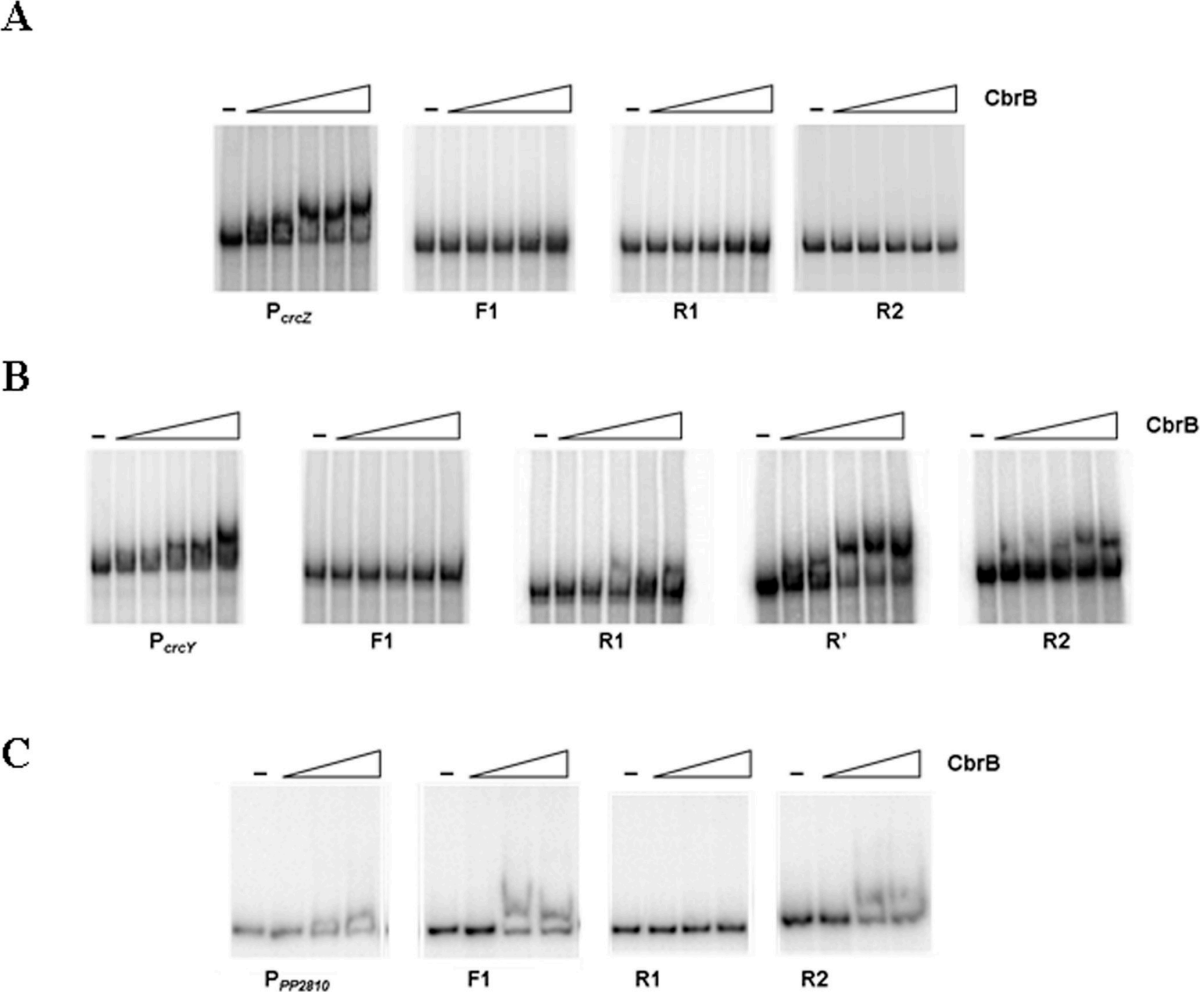
Electrophoretic mobility shift assay (EMSA) for CbrB binding. Band shift observed in linear dsDNA fragments containing the promoter regions of *crcZ* **(A)**, *crcY* **(B)** and *PP2810* **(C)** and the substituted versions of F1, R1, (R’) or R2 for each promoter, in the presence increasing amounts of CbrB (0, 0.38, 0.75, 1, 1.5 and 2 μM for *crcZ* and *crcY* promoters and 0, 1, 2 and 2.4 μM for *PP2810*). A representative assay of three replicas performed is shown.

The binding affinity of CbrB to DNA fragments containing the wild type sequences of the *crcZ*, *crcY* or *PP2810* promoters, and their substituted versions of F1, R1, R’ or R2 was analysed in EMSA assays (Fig 4). First, the affinity of CbrB to the promoter region of PP2810 resulted lower to that of *crcZ* and *crcY*, which is consistent with a lower expression levels, especially in less carbon-limited conditions such as succinate or LB.

Substitution of any of the subsites F1, R1 or R2 for P_*crcZ*_ (which are perfect matches in sequence) resulted in an inability of CbrB to bind to the DNA fragment, although they retained partial activity for transcriptional activation with two conserved subsites (Fig 3). This fact shows that *in vitro* binding is most sensitive to a change in the equilibrium of interactions of CbrB with the DNA, than *in vivo* activation. Substitution of F1 in P_*crcY*_ prevented CbrB binding, while the substitution of R1 slightly reduced its binding affinity. Alteration of R’ and R2 did not significantly affect the binding affinity of CbrB to P_*crcY*_. In the case of PP2810, the only sequence that prevented CbrB binding was R1, which is the most similar to the consensus, and substitution F1 or R2 (poorly conserved) did not cause any obvious alteration.

Interestingly, alteration of the putative CbrB-binding subsites in the targets *crcY* and *PP2810* had a parallel effect on both expression and binding to the DNA. On the other hand, binding of CbrB to P_*crcZ*_ is somehow more sensitive, and alteration of any of the three subsites prevented CbrB binding. However, due to the high efficiency in transcriptional activation from the *crcZ* promoter, the effects on transcription of the altered F1 and R1 sequences are only evident under conditions of lowest CbrB activity. Altogether, these results suggest that a clear preferential binding subsite for CbrB cannot be defined but rather, there is an accumulative effect of each one, and their relative contribution depend on the target region.

**Fig 5:**
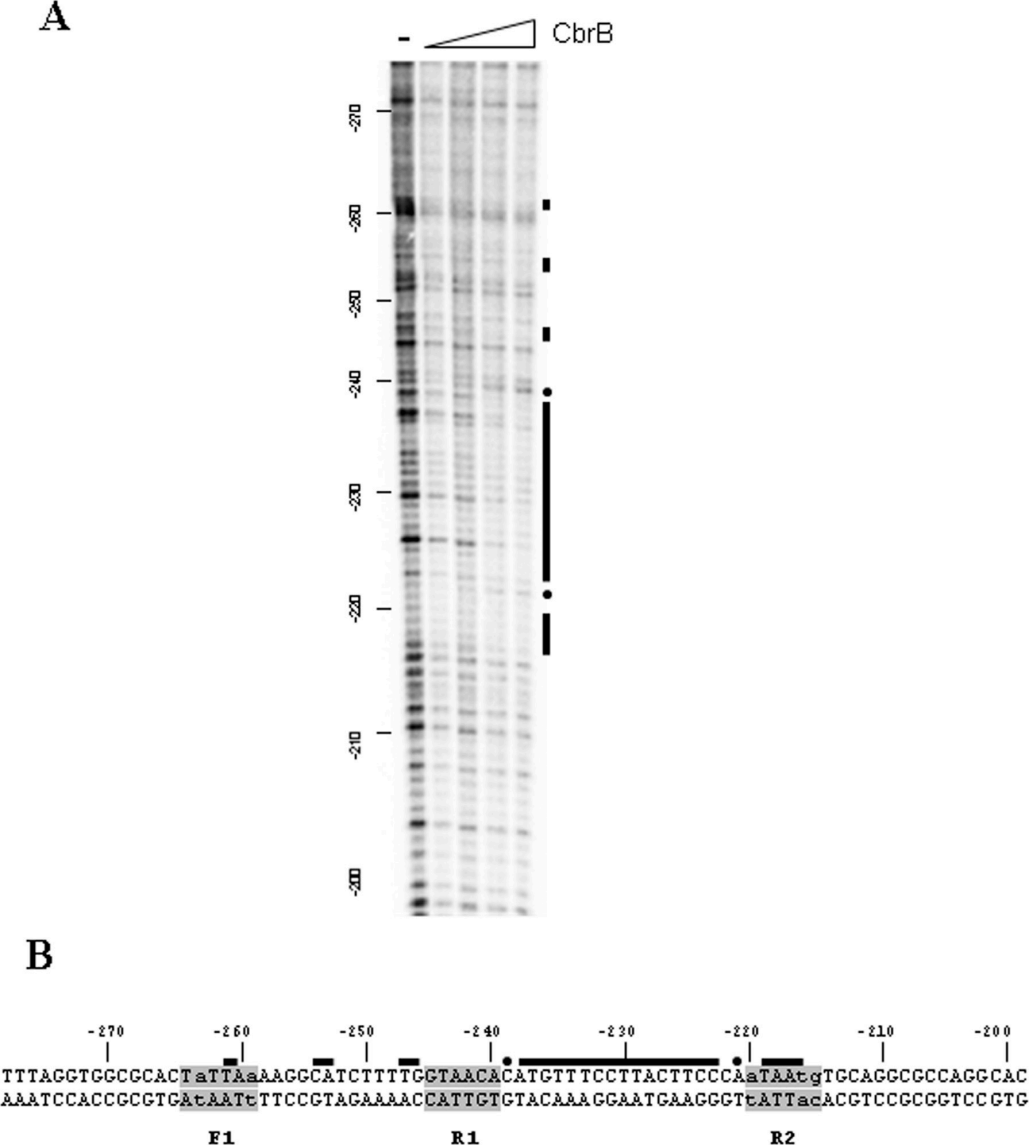
DNase I footprint of CbrB on the *PP2810* promoter region. **(A)** CbrB footprint analysis for a linear fragment containing the promoter region of *PP2810* with increasing concentrations of CbrB. CbrB- protected regions to DNAse I digestion (black bars), and hypersensitive positions (dots) are marked. The coordinates are relative to the ATG. **(B)** Schematic representation of the CbrB-protected regions on the sequence of the *PP2810* promoter region, with the same references as for the footprint pattern. Shaded boxes on the *PP2810* promoter sequence indicate proposed CbrB- binding subsites.

In addition, the precise interaction of CbrB with the DNA promoter region of *PP2810* was examined by DNase I footprinting analysis. The direct interaction of CbrB with the promoter region revealed some protected areas and also regions of hypersensitivity to DNAse I digestion, thus suggesting distortion of the DNA in this area upon CbrB binding (Fig 5). The altered digestion pattern was detected between sites F1, R1 an R2 and resembled the ones of *crcZ* and *crcY* as revealed by the continuous protection between the subsites [7]. However, the lower binding affinity to PP2810 allowed detection of a very weakly altered pattern in the most upstream subsite and did not allowed binding detection at all in the lower strand of the DNA. Overall, the DNAse I digestion arrangement in the region covering positions −210 to −270 of the promoter region indicated an alteration in the topology of the DNA helix upon CbrB binding, and unequivocally showed a direct CbrB binding and consequently regulation of the PP2810-13 operon.

### CbrB is a monomer in solution

**Fig 6:**
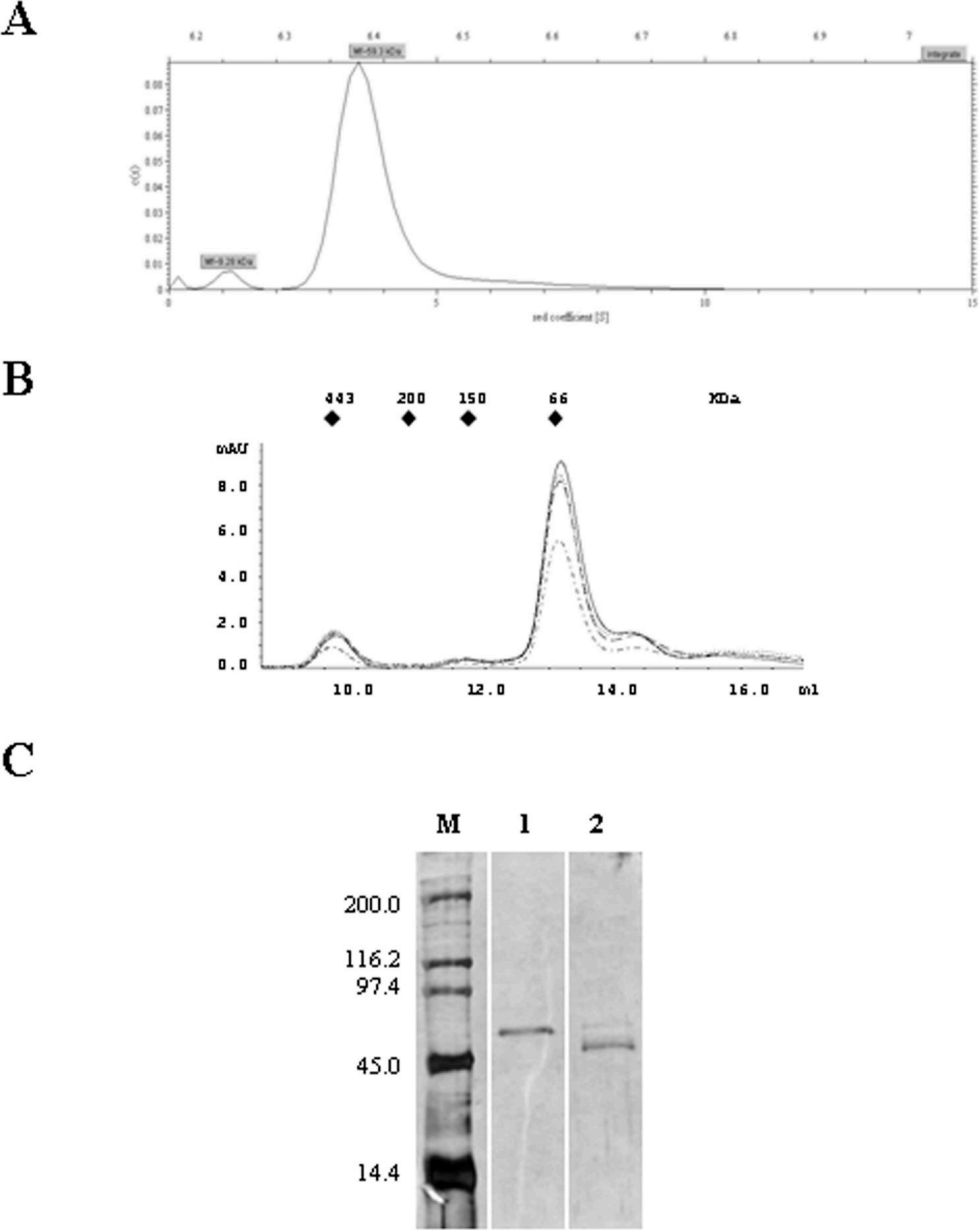
Size-distribution analysis of CbrB. **A)** Sedimentation velocity ultracentrifugation chromatogram and calculated differential sedimentation coefficients for each form. **B)** Analytical gel filtration by Molecular Exclusion Chromatography of CbrB. The chromatograms corresponded to 2 μM CbrB (solid line, ___), CbrB treated with acetyl phosphate (dashed and dotted line, .__.__), incubated in the presence of a 72 pb fragment containing the *crcZ* promoter region (dotted line, ….) or in the presence of acetyl phosphate and a DNA fragment containing the *crcZ* promoter (dashed line, __ __). The Molecular weight markers are represented as blue triangles at the top of the chromatogram (Apoferritin, 443 KDa, β- Amylase, 200 KDa, alcohol dehydrogenase, 150 KDa and bovine serum albumin, 66 KDa). **C)** SDS-PAGE acrylamide gel containing the fraction of high molecular weight from Molecular exclusion chromatography of CbrB (line 1), and the pure CbrB preparation (line 2). Molecular weight marker contains Myosin, β-galactosidase, Phosphorylase b, ovoalbumin, lysozyme (200, 116.2, 97.4, 45.0 and 14.4 KDa, respectively). Full-length gel of Fig 6C is presented in S4 Fig.

The fact that the CbrB binding site does not contain palindromic sequences suggests that CbrB might not bind as a dimer and, therefore, it should not be a dimer in solution. Ultracentrifugation of CbrB was performed in order to determine the aggregation status of the protein. The analysis of the CbrB sedimentation profile showed a main peak with a sedimentation coefficient to water at 20°C (s20, w) of 3.76 S. The slight asymmetry seen to the right of the peak indicates the existence of certain aggregation of the sample (Fig 6A). The molecular mass estimated for the main species is 59.3 KDa, which corresponds to a monomer of CbrB in solution (predicted by sequence to be 53.4 KDa). The molecular weight of the major fraction of CbrB was also coincident with the size expected for a monomer in solution when analysed by molecular exclusion chromatography (Fig 6B, solid line). A very small fraction that could correspond to a dimer was detected, eluting just before the monomer, but had insignificant representation. In addition, a fraction of larger size that eluted at a volume close to 10 ml, which could correspond to higher order complexes of CbrB was also detected. The fraction corresponding to a high molecular weight form was run in a denaturing SDS polyacrylamide gel (Fig 6C, line 1) together with the pure CbrB preparation (Fig 6C, line 2) and showed a slightly larger size than the monomer of CbrB, so it was analysed by mass spectrometry. The results showed that the fraction corresponded to a bifunctional polymyxin resistance protein ArnA of *Escherichia coli* with a Mascot protein score [22] of 146, thus confirming that the protein apparently co-eluted as a contaminant with the His-tagged CbrB in the Cobalt resin during the purification process, and was discarded to be a multimeric form of CbrB.

Since the binding sites in the three identified targets (*crcZ*, *crcY* and *PP2810*) are not palindromic, and CbrB is mainly detected as a monomer in solution, we aimed to determine if in the presence of a DNA containing the CbrB binding sites or the phosphorylation state of CbrB could promote the multimerization of the protein. Addition of a 72 bp DNA fragment containing the subsites F1, R1 and R2 of the promoter region of *crcZ* to the CbrB sample in the size exclusion chromatography did not change this pattern. Similarly, CbrB treatment with acetyl phosphate in an attempt to phosphorylate it neither changed this pattern (Fig 6B).

## Discussion

While the CbrAB two-component system has been widely described in the assimilation of different amino acids as carbon sources [6, 12], how the bacterium senses these signals to activate the system is largely unknown. Expression analysis revealed its implication in many cellular processes and positioned the regulatory system in a high ranked hierarchy of regulation. Also, the fact that CbrB also indirectly controlled Carbon Catabolite Repression mediated by Crc in the Pseudomonads through transcriptional activation of the regulatory RNAs CrcZ and CrcY [8, 9, 23], made necessary to define the particular targets contacted by CbrB and to discriminate between regulons. CbrB binding to DNA promoter sequences containing σ^N^ consensus sites was analysed and, for the first time, we show direct activation by CbrB (in addition to CrcZ and CrcY regulatory RNAs) of new target genes coding for proteins. The fact that an important number of regulatory and signal transduction proteins are among the potential targets for CbrB regulation (20% of the targets were involved in regulatory processes), indicates that the CbrB regulon could be very large. On the other hand, 38% of the CbrB targets were annotated as hypothetical proteins that limited the interpretation of its CbrB-mediated control. One of these targets, operon *PP2810-PP2013*, has structural homology to an RND- type efflux pump that is activated in situations of carbon limitation according to our results. Its promoter arrangement is coherent with the disposition of the CbrB, IHF and σ^N^ consensus binding sites found in the *crcZ* and *crcY* promoters, and the fact that it is a target of different nature than non-translated regulatory RNAs, led us to use it for its dissection in this work.

We have identified 61 targets from the ChIP-Seq analysis that were selected under very restrictive conditions with the purpose to determine a DNA binding sequence when CbrB acts as transcriptional activator of σ^N^-dependent promoters. We are aware that many targets with less recognizable σ^N^ sites could be overlooked. In this sense, it has been reported that poor σ^N^ sequences are tightly regulated by Enhancer Binding Proteins (EBPs) when IHF contributes to adopt the appropriate topology for the formation of the initiation complex [16, 24]. On the other hand, the fact that 20% of the targets are involved in regulatory processes, amplifies considerably the regulon in cascade.

Also, some EBPs of this family such as NtrC or FleQ, which usually activate transcription of σ^N^ promoters have been reported to occasionally act as repressors of the transcription of some genes with σ^70^ promoter sequences [25–27]. This particular scenario has not been evaluated in this work and only fragments peaks containing σ^N^-dependent promoters were included. Nevertheless, three of the target genes validated by quantitative RT-PCR resulted to be repressed (*oprD*, *rpoN* and *glgA*). Although sequences for CbrB binding were identified in the DNA fragment obtained from the ChIP-Seq analysis, its disposition with respect to the putative σ^N^ promoter was incoherent, so our interpretation is that their transcription is not dependent on this sigma factor.

Bioinformatics analysis using MEME or FIMO (MEME suite, NIH) [28] on the promoter regions of the selected peaks did not reveal a defined pattern for CbrB binding, since 6 bp imperfect sequences with 1 to 2 mismatches occur recurrently in random sequences. The application of a more restrictive custom algorithm in the DNA fragments, scanning for at least two of the subsites (F1, R1 or R2) with a proper spacing, and allowing up to 2 mismatches overall, also failed to generate a consensus sequence for CbrB binding.

Also, two of the targets from the analysis resulted to be not CbrB -regulated, although PP3009 even contained a potential CbrB binding site. This might mean that CbrB, as activators having a short recognition sequence such as NifA that recognises a single TGT-N_10_-ACA site [29], could bind to many more sequences in the genome than those used to activate transcription. As for CbrB, NifA activation is IHF-dependent, and their association to promoter sequences in the right positions causes the appropriate interaction with the RNA polymerase facilitated by IHF, leading to transcription activation. CbrB dependence on IHF might also explain why a number of potential CbrB binding sites could not be optimal at all but still be functional, when in combination with a good promoter sequence and the interplay of IHF facilitating the interaction of the transcription factors and consequent open complex formation.

The lack of a restricted consensus sequence for CbrB binding to make an *in silico* prediction together with the mutational analysis in the CbrB subsites that reveal the different relevance of each one according to the context, suggests that CbrB may bind to the DNA in a relatively relaxed (variable) manner. The participation of IHF may contribute to the contextualization to favor CbrB activation in sites with different affinities.

The multimeric state of CbrB also seems to play an important role in the mechanism of activation. A typical enhancer binding protein contains three domains: an N-terminal regulatory domain, a central AAA+ domain and a C-terminal DNA-binding domain [30]. The N-terminal domain regulates the central AAA domain activity either positively or negatively, often through the control of oligomerisation (reviewed in [18, 31]). EBPs are typically dimeric in their inactive state but in response to stimulatory signals, form higher order oligomers via their AAA+ domain. Hexamerisation is required both for ATPase activity and for interaction with the holoenzyme bound at the promoter and remodelling the closed complex into a transcriptionally competent open complex (reviewed in [18, 32]). Stable hexamers have been observed for PspF, NtrC, NtrC1 and ZraR in the presence of different nucleotides implying that nucleotides play significant roles in oligomerisation (reviewed in [32, 33]). While multiple Upstream Activating Sequences (UAS) are common in σ^N^-dependent promoters probably to facilitate oligomerization of contiguously bound dimers, an absolute dependency on more than one target site is unusual, and in some cases, such a NifA, a single palindromic sequence is found in its binding sites. Intriguingly, this is the case of NorR where three inactive NorR dimers bind to three adjacent UAS, resulting in an increased local concentration of NorR thus facilitating oligomerisation upon activation of the regulatory GAF domain[34]. For CbrB, lack of palindromic sites and no perceptible dimerization *in vitro* suggest that it does not bind as a dimer. Rather it appears to initially bind as a monomer to each one of the sites. Although there is no evidence of oligomerisation of CbrB in solution, the formation of structures of higher order is the most rational prediction to stabilise the complex *in vivo*. Each particular binding site has an accumulative effect upon CbrB binding, although neither of the three is essential for transcriptional activation. Actually, one of the subsites is almost dispensable at the *crcY* and *PP2810* promoter regions; only at *crcZ*, where there is a higher conservation of the sites and higher level of expression, a greater participation of each UAS is observed, although there is still a substantial activation after mutating any of the subsites. As shown for *PP2810*, even though the subsites at the promoter region are less conserved, the proper spacing of the UAS is preserved and its expression is tightly regulated (Fig 1B), suggesting that it may be even more relevant than a high conservation of the sequences. Proper spacing of the subsites places them on the same side of the DNA helix, favoring the interaction of CbrB with DNA at the same time as the contacts among CbrB monomers.

The work presented here pictures a landscape on the complexity of the binding recognition mechanism of CbrB to its DNA targets, which include three important elements; CbrB binding to the DNA contacting at least two non-palindromic subsites of variable spacing and low sequence conservation, the participation of IHF to favor CbrB interaction at certain sites and finally the presence of a σ^N^ sequence for specific activation. This panorama opens a new door for further research on the novel functions controlled by this regulator.

## Materials and methods

### Bacterial strains and growth conditions

The bacterial strains, plasmids and oligonucleotides used in this work are summarized in S1 and S2 Tables. Cells were grown in M9 minimal medium[35], containing 20 mM sodium succinate or oxaloacetate as carbon source and ammonium chloride (1 g l^−1^) as the nitrogen source. Luria–Bertani (LB) was used as a rich medium[36]. Cultures were grown in culture tubes or flasks with shaking (180 r.p.m.) at 30°C and 37°C for *Pseudomonas* or *E*. *coli* strains, respectively. When required, antibiotics and other additives were used at the following concentrations (μg ml^−1^): ampicillin (Ap) 100; carbenicillin (Cb) 500; kanamycin (Km) 25; rifampicin (Rf) 20 and 5-bromo- 4-chloro-3-indoyl-β-D-galactopyranoside (X-Gal) 25. All reagents were purchased from Sigma-Aldrich.

### Plasmid construction

All DNA manipulations were made using standard protocols[36]. Plasmid DNA preparation and DNA purification kits were purchased from Macherey-Nagel, Promega and General Electric Healthcare according to the manufacturers’ specifications. In all cloning procedures involving PCR amplification, sequences of the amplified fragments were determined by commercial sequencing (Secugen, Madrid, Spain). *E*. *coli* DH5α was used as a host in all cloning procedures. Plasmid DNA was transferred to *E*. *coli* and *P*. *putida* strains by transformation[37] or electroporation[38], respectively.

Plasmid pMPO389 was generated by PCR amplification of genomic DNA using the pair of oligonucleotides crcYSmaI/ crcYBamHI into a BamHI-SmaI digested pUC18Sfi vector. The transcriptional fusions of *PP2810* promoter region to *lacZ* were constructed by PCR amplification of *P*. *putida* KT2442 genomic DNA with oligonucleotides PP2810F/PP2810R and PP2810fwmut/PP2810R cloned into the EcoRI- and BamHI-digested vector pMPO234 to give plasmids pMPO420 and pMPO422 respectively. The substitutions of the wild type sequences of F1, R1 and R2 for CbrB binding subsites were generated by PCR extension overlapping fragments as in [39] using *P*. *putida* KT2442 genomic DNA as the template and the mutagenic oligonucleotides PP2810mutIF/ PP2810mutIR for F1, PP2810mutIIF/ PP2810mutIIR for R1 and PP2810mutIIIF/ PP2810mutIIIR for R2, with the external nonmutagenic oligonucleotides PP2810R and PP2810fwmut. The final PCR products were cloned as EcoRI/BamHI in pMPO234 to yield pMPO425, pMPO426 and pMPO428, respectively.

The transcriptional fusions to *lacZ* containing the substituted F1, R1 and R2 CbrB binding subsites for the promoter regions of *crcZ* were constructed using pMPO1316 as template and the mutagenic oligonucleotides Zmut1F/Zmut1R, Zmut2F/Zmut2R, Zmut3F/Zmut3R, respectively with the external non-mutagenic oligonucleotides ZbisBam/ fuscrcZFEco. The final PCR products were cloned as an EcoRI/BamHI fragment into pMPO234 to yield pMPO436, pMPO437 and pMPO438, respectively. In the case of the promoter region of *crcY*, plasmid pMPO1314 was used as the template and the mutagenic oligonucleotides Ymut1F/ Ymut1R were used to substitute F1, Ymut2F/ Ymut2R for R1, Ymut3F/ Ymut3R for R’ and Ymut4F/ Ymut4R for R2 site. The external non-mutagenic oligonucleotides to generate the overlapping mutated fragment were FusioncrcYRbisBam and crcYSmaI for the fragment bearing the mutated F1 and FusioncrcYRbisBam and FusioncrcYF for the fragments containing the mutated R1, R' and R2. After EcoRI/BamHI directed cloning into pMPO234, plasmids pMPO439, pMPO440, pMPO441 and pMPO442 were generated, respectively. Plasmids pMP1342 and pMPO1343 were constructed by PCR amplification of genomic DNA using the pair of oligonucleotides foot2810smaF+/ foot2810XbaR+ and foot2810XbaF-/ foot2810smaR- for the top and bottom strands of the DNA respectively, and the fragments were cloned into a XbaI–SmaI digested pUC18Sfi vector.

### ß-Galactosidase assays

Steady-state β-galactosidase assays were used to examine the expression of the P_*crcZ*_, P_*crcY*_ and P_*PP2810*_ transcriptional fusions to *lacZ* and their mutagenized versions in *P*. *putida* KT2442 and MPO401 under different growth conditions. Preinocula of bacterial strains harbouring the relevant plasmids were grown to saturation in LB and minimal medium with succinate 20 mM as carbon source. Cells were then diluted to 0.05 of A_600_ in LB, to 0.1 of A_600_ in a minimal medium with succinate and to 0.3 of A_600_ in a minimal medium with oxaloacetate and shaken until they reached the mid-exponential phase (A_600_ = 0.25–0.5). β-galactosidase activity was determined from SDS- and chloroform-permeabilised cells as previously described[40]. All plasmids in *E*. *coli* were transferred by conjugation to *P*. *putida* in the presence of the helper vector pRK2013.

### Protein purification and Molecular exclusion chromatography

CbrB was overexpressed and purified as previously described [7]. CbrB Molecular Exclusion chromatography was performed on a Superdex 200 increase 10/300GL column (GE Healthcare) in an ÄKTA FPLC system (GE Healthcare). Chromatography was performed with 500μl of 2μM CbrB at room temperature in CbrB binding buffer (50 mM Tris HCl pH 7.4, 50 mM KCl, 0.1 mM DTT, 0.1 mM EDTA and 10 mM MgCl2) with a flow rate of 0.5 ml min^-1^. CbrB samples were incubated in presence/absence of 4.5 nM 80pb-DNA fragment containing the *crcZ* promoter region and/or 80 mM acetyl phosphate as in [7] where needed prior to loading onto the column. An 80 bp DNA fragment lacking any CbrB binding sites was used as negative control. Apoferritin (443 KDa), β- Amylase (200 KDa), alcohol dehydrogenase (150 KDa) and bovine serum albumin (66 KDa) were used as Molecular Weight markers. The eluted fraction corresponding to a high molecular weight from the molecular exclusion chromatography was analysed by Mass spectrometry at the University Pablo de Olavide Central Services.

### Analytical ultracentrifugation

Sedimentation velocity experiments were carried out at 20 °C in an Optima XL-A analytical ultracentrifuge (Beckman Coulter) at Instituto de Química-Física Rocasolano, CSIC, Madrid. Measurements were performed in CbrB binding buffer at 45,000 rpm using a protein concentration of 60 μm. Differential sedimentation coefficients c(s) were calculated by least squares boundary modelling of sedimentation velocity profiles using the program sedfit [41].

### Electrophoretic mobility shift assay (EMSA)

Electrophoretic mobility shift assays of the CbrB-DNA complexes were performed as previously described [7]. DNA probes comprising the CbrB binding sites were obtained as dsDNA fragments by annealing of 72 pb oligonucleotides containing the wild type sequences or substitutions of subsites F1, R1, R’ or R2 for P_*crcZ*_ and P_*crcY*_ promoter regions (see S2 Table), and by PCR amplification and XbaI/ SmaI restriction of the products for the remaining genes. The probe for PP2810 promoter region and their derivatives containing substitutions of F1, R1 and R2 CbrB binding subsites were obtained by PCR amplification with oligonucleotides PP2810EMSAXbaIF/ PP2810EMSASmaR using plasmids pMPO420, pMPO425, pMPO426 and pMPO428 as templates, respectively. The promoter regions of PP1206 and PP3420 were obtained by XbaI/SmaI restriction digest of PCR fragments obtained by amplification of genomic DNA with the oligos EMSAoprDF/ EMSAoprDR and EMSA3420HKF/ EMSA3420HKR, respectively. The probes containing the promoter regions of *crcZ* and *crcY* were labelled with [α-^32^P]-dATP and all the other fragments were labelled with [α-^32^P]-dCTP by filling in the 5′ overhangs using the Klenow fragment. Reactions were performed in 20 μl at room temperature in Crc binding buffer (35mM Tris-acetate pH 7.9, 70 mM KAc, 20 mM NH4Ac, 2 mM Mg_2_Ac, 1mM CaCl_2_, 1 mM DTT, 5% glycerol) for *crcZ* and *crcY* or CbrB binding buffer (50mM Tris-HCl pH 7.4, 50 mM KCl, 10 mM MgCl_2_, 0,1 mM EDTA, 1 mM DTT) for the others reactions. The reactions were performed as in [7] with the DNA probe (5 nM for *crcZ* and *crcY* and 1.5 nM for the remaining promoters), 100 ng μl^−1^ salmon sperm DNA, 250 ng μl^−1^ BSA and increasing amounts of purified protein CbrB (see Fig legends in Figs 1A and 4) for *PP2810*, *oprD*, *PP3420*, *crcZ* and *crcY* promoters.

### DNAse I footprinting

CbrB DNase I footprinting was performed essentially as described[7]. The DNA fragments were generated by XbaI/SmaI restriction digest of pMPO1342 and pMPO1343 containing the promoter region of PP2810 for the top and bottom strands. The DNA fragments were labelled with [α-^32^P]dCTP by filling in the 5′ overhangs using the Klenow fragment. The binding reactions were performed in 20 μl at room temperature in the same conditions as the EMSA with CbrB at growing concentrations of 0, 1, 2, 4 and 4.9 μM. A sequencing reaction performed with the Sequenase 2.0 kit (USB) using an oligonucleotide specific for the labelled strand in each case (sec2810cad- and sec2810cad + for the top and bottom strands of *P*_*PP2810*_) and was run with the partially digested DNA used as a size marker.

### RNA preparation and Quantitative RT-PCR

*P*. *putida* KT2442 or its derivative MPO401 was cultivated in LB or minimal medium with succinate as carbon source. The saturated preinocula were diluted to A600 = 0.05 in LB, and 0.1 in a minimal medium with succinate from saturated cultures in the same medium and grown to mid-exponential phase. Since the strains did not grow with OAA as carbon source, the cultures were inoculated at A600 = 0,3 and were induced in the medium for 4 h, then collected and frozen at -80°C. RNA extraction was prepared as described [7]. Quantitative reverse transcription (RT)-PCR of the expression of the candidates was performed as described previously [42]. RT of 6 μg of total RNA was carried out using the High-Capacity cDNA Archive Kit (Applied Biosystems), with random hexamers as primers to generate cDNAs. Target cDNAs (1–25 ng) from the experimental samples were amplified in quadruplicate in separate PCR reactions using 0.3 mM of each primer (crcZ49-1Q/ crcZ99-2Q for *crcZ*, QflgBF/ QflgBR for PP4391, QPP0952F/ QPP0952R for PP0952, QPP1206F/ QPP1206R for PP1206, QPP3009/ PP3009_for PP3009, QPP3074F/ QPP3074R for PP3074, QPP3420F/ QPP3420R for PP3420, QPP3656F/ QPP3656R for PP3656, QPP4050F/ QPP4050R for PP4050, QPP4486F/ QPP4486R for PP4486 and QPP4643F/ QPP4643R for PP4643) as previously described [7].

### Chromatin immunoprecipitation with high-throughput sequencing (ChIP-Seq)

Three biological replicates of 250 ml batch cultures of *P*. *putida* KT2442 were independently grown up to mid-exponential phase in a minimal medium containing oxaloacetate as carbon source from pre-culture grown on succinate at 30°C. 1/10 volume of fresh crosslinking solution (50 mM Hepes–KOH, 100 mM NaCl, 1 mM EDTA, 0.5 mM EGTA, 11% formaldehyde) was added and incubated at room temperature for 15 min. The reaction was stopped by the addition of 1/20 volume of 2.5 M glycine, and the cells were swirled briefly and incubated at room temperature for 5 min. Cells were rinsed twice with ice cold PBS, and collected at 2000 rcf for 10 min and frozen at -80°C. The pellet was resuspended in 10 ml of Lysis Buffer (50 mM Hepes–KOH, pH 7.5; 140 mM NaCl; 1 mM EDTA; 1% Triton X-100, 0.1% sodium deoxycholate and 1 mM PMSF), and sonicated with a Bioruptor® UCD-200 (Diagenode) in an ice-water bath in 6 cycles of 30 sec on/off for 15 min, until DNA was sheared up to 200–300 bp fragments. For CbrB-DNA bound immunoprecipitation, 80 mg of CbrB antibodies coupled to Protein A Dynabeads (Invitrogen) in washing buffer (PBS pH 7.4, 0,02% Tween 20) following the manufactures’ indications. The complex was incubated overnight at 4°C at a rotatory shaker for specific binding. The antibodies were removed by immobilization of the magnetic beads and after washing twice, the supernatant was removed for antigen elution by denaturation. The extract was resuspended in 40 μL of elution buffer (50 mM TrisHCl pH8, 10 mM EDTA, SDS 1%) and incubated at 65°C for 15 min. Then, the supernatant was incubated for 30 min with RNaseA (50ug/mL) at 37°C and de-proteinised with proteinase K (0.4 mg/mL), incubated for 2 hours at 37°C and further inactivated at 55°C for two hours. DNA was purified with the QIAquick PCR Purification Kit (QIAquick spin kit, QIAGEN), the sample was eluted in 100 μl of mQ H_2_O and stored at -20°C.

Sequencing libraries were prepared using the Ion ChIP-Seq library preparation kit for PGM (Thermo Fisher Scientific Inc). Ten ng of IP DNA was used as input. Bi-directional 200 bases sequencing was perform using a PGM Ion-Torrent system in Genomic´ s Core Facility at Cabimer. Two technical replicates were sequenced and quality was verified by a FastQC analysis (Babraham Bioinformatics). Reads were aligned on *P*. *putida* KT2440 using TMAP 4.4.23–1 with Torrent Suite (version 4.4.3) from PGM. Data were uploaded to the Galaxy Web platform to use the public server at usegalaxy.org [43] and BAM files were merged together with BAM tools [44] for further analysis. Peak detection was performed using MACS2 (Model-based analysis of ChIP-Seq version 2.0.10) to identified enriched regions in gene promoter regions (Band width of 300bp; P-value <0.01; shift size of 100bp and MFOLD lower limit 10-upper limit 30). Peaks were visualized with Integrative Genomics Viewer (Broad Institute). Sequences were deposited in the database of NBCI with SRA accession number SRP158108.

## Supporting information

S1 Fig Captures of the ChIP-seq raw data in a IGV browser. In cyan the total peaks after peak calling, in green the peaks with a p-value<2, in red the selected DNA fragments after the σN filtration used for the search of a CbrB binding consensus sequence. Bam files representing the reads are shown below.
(TIF)

S2 Fig Electrophoretic mobility shift assay (EMSA) for CbrB binding. Linear dsDNA fragments containing the promoter regions of PP4391, PP3074, PP4050 in the presence increasing amounts of CbrB (indicated on top) were used. In parenthesis the coordinates of the DNA fragments used for the EMSA referred to the ATG.
(TIF)

S3 Fig *In vivo* expression from the *P. putida crcZ* and *crcY* promoters. Expression was measured as β-galactosidase activity of the *crcZ* (A) and *crcY*::*lacZ* (B) transcriptional fusions (plasmid pMPO1316 and pMPO1314, respectively) of cultures of the KT2442 wild-type and the MPO401 (Δ*cbrB*) mutant strains, grown in LB (white bars) or in minimal medium containing succinate (black) or oxaloacetate (grey bars) as carbon sources. The values are the average of at least three independent assays. The error bars indicate the standard deviation of the means. Stars designate p-values for the Student's t-test for unpaired samples not assuming equal variance and are referred to the wild type strain. *: p<0.05; **: p<0.01; ***:p<0.005.
(TIF)

S4 Fig Full-length gel of Fig 6C. (TIF)

S1 Table Strains and plasmids used in this study. (PDF)

S2 Table Oligonucleotides used in this study. (PDF)
