# Supplementary figures and images for: The CbrB Regulon: Promoter dissection reveals novel insights into the CbrAB expression network in Pseudomonas putida
Source: PLoS One. 2018 Dec 17;13(12):e0209191. doi: 10.1371/journal.pone.0209191 (PMC6296734; doi:10.1371/journal.pone.0209191)

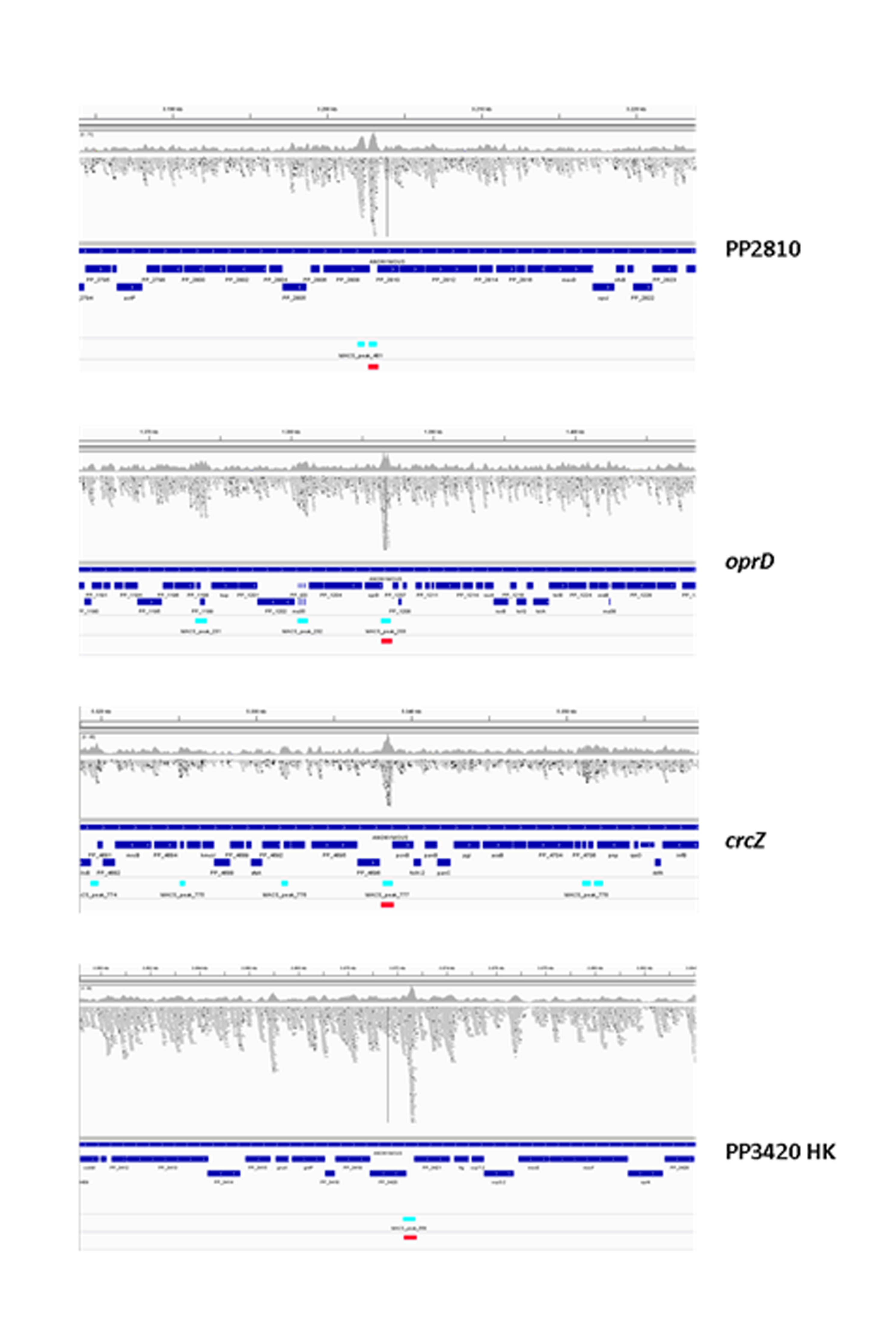

Supplement: S1 Fig — In cyan the total peaks after peak calling, in green the peaks with a p-value<2, in red the selected DNA fragments after the σN filtration used for the search of a CbrB binding consensus sequence. Bam files representing the reads are shown below. (TIF) [file pone.0209191.s001.TIF]

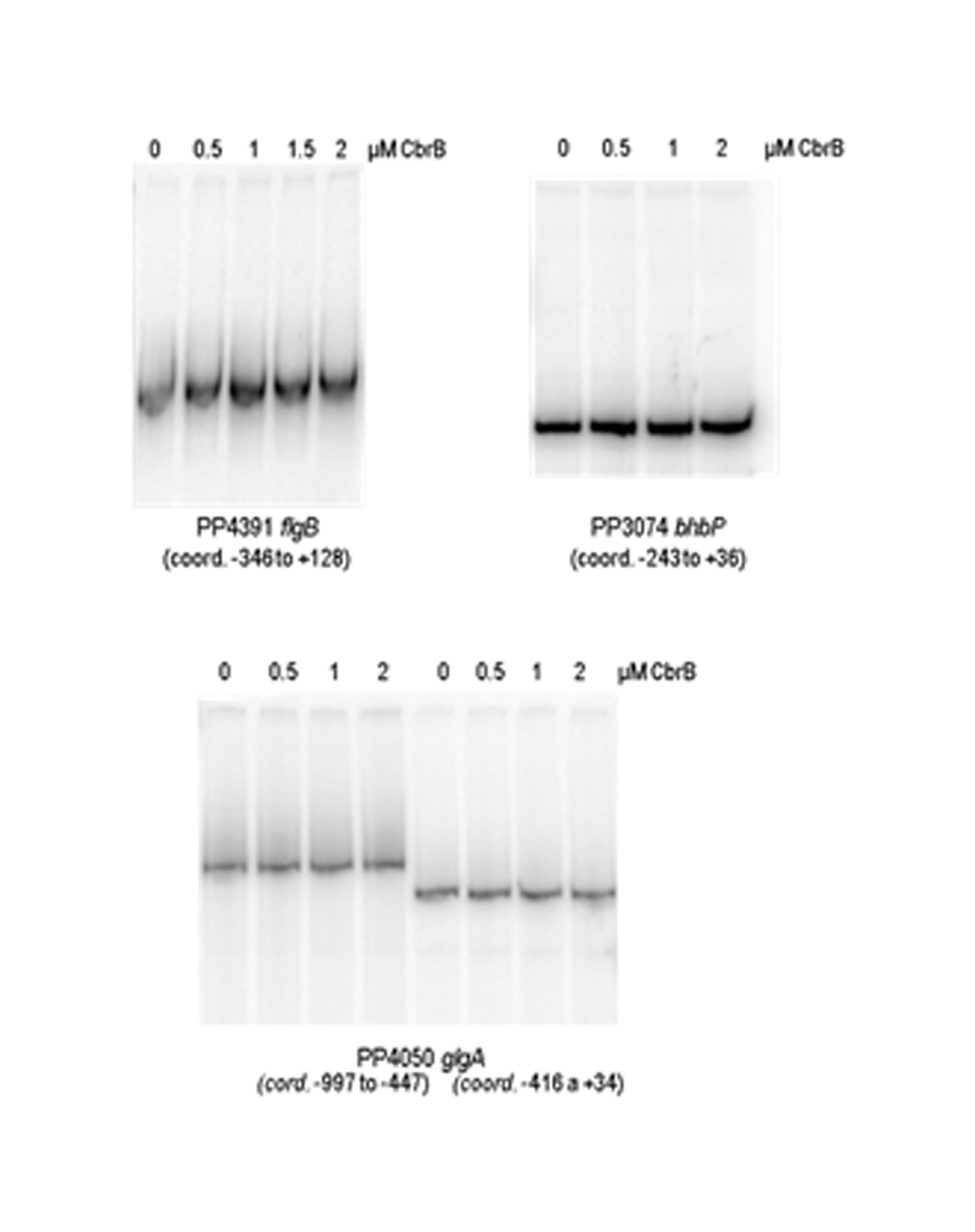

Supplement: S2 Fig — Linear dsDNA fragments containing the promoter regions of PP4391, PP3074, PP4050 in the presence increasing amounts of CbrB (indicated on top) were used. In parenthesis the coordinates of the DNA fragments used for the EMSA referred to the ATG. (TIF) [file pone.0209191.s002.TIF]

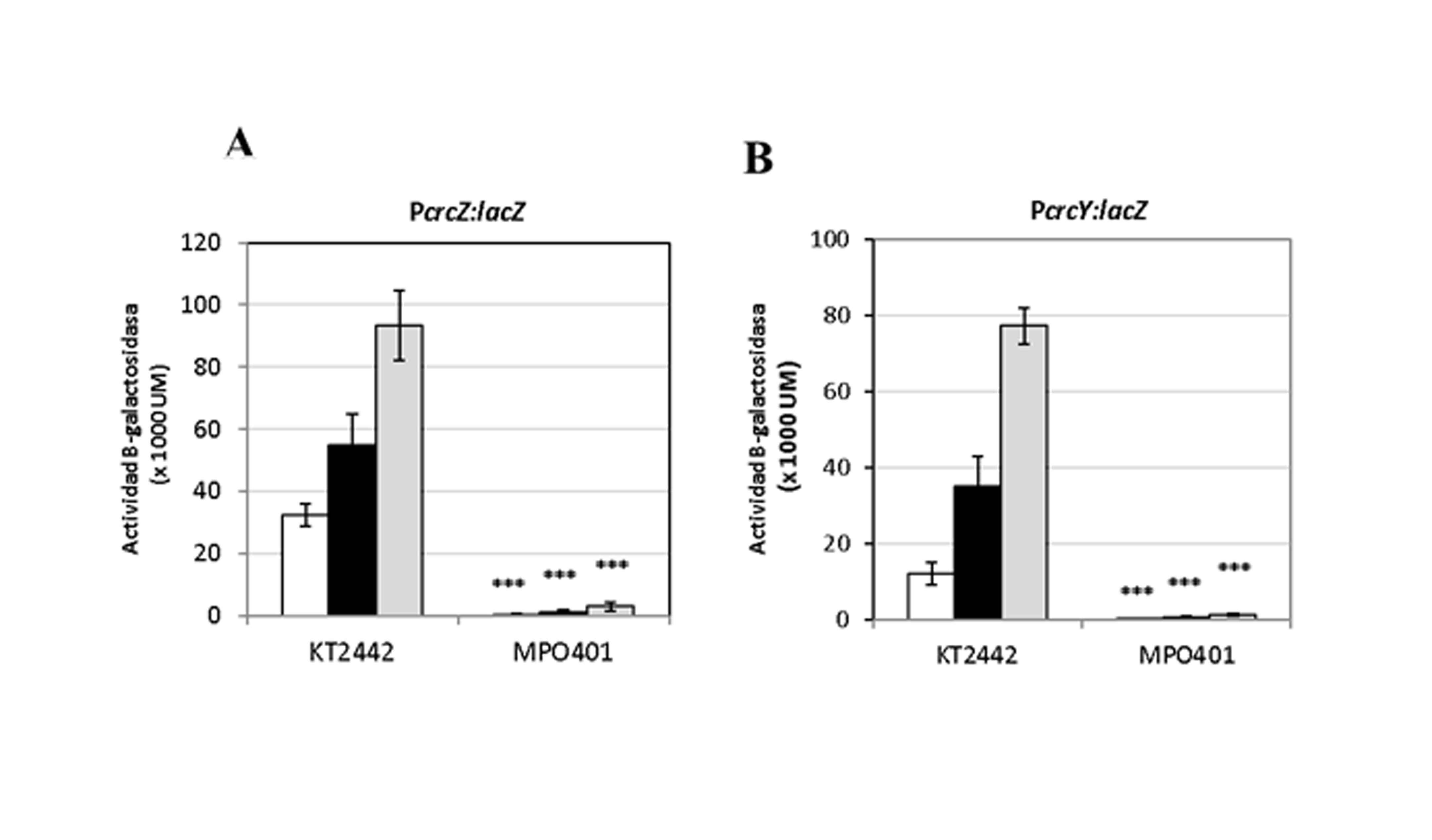

Supplement: S3 Fig — Expression was measured as β-galactosidase activity of the crcZ (A) and crcY::lacZ (B) transcriptional fusions (plasmid pMPO1316 and pMPO1314, respectively) of cultures of the KT2442 wild-type and the MPO401 (ΔcbrB) mutant strains, grown in LB (white bars) or in minimal medium containing succinate (black) or oxaloacetate (grey bars) as carbon sources. The values are the average of at least three independent assays. The error bars indicate the standard deviation of the means. Stars designate p-values for the Student's t-test for unpaired samples not assuming equal variance and are referred to the wild type strain. *: p<0.05; **: p<0.01; ***:p<0.005. (TIF) [file pone.0209191.s003.TIF]

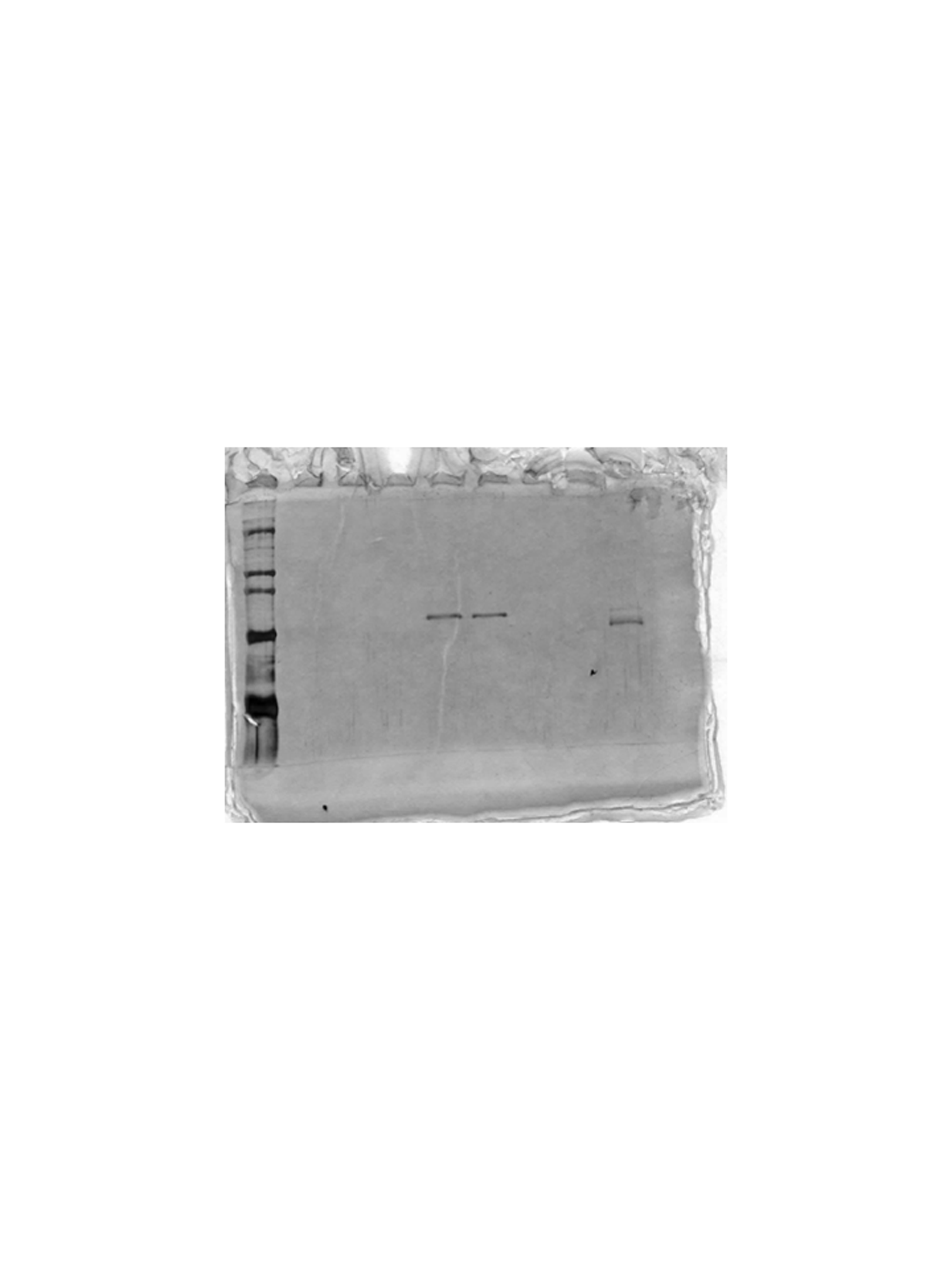

Supplement: S4 Fig — (TIF) [file pone.0209191.s004.TIF]
