# Supplementary material for: The CbrB Regulon: Promoter dissection reveals novel insights into the CbrAB expression network in Pseudomonas putida
Source: PLoS One. 2018 Dec 17;13(12):e0209191. doi: 10.1371/journal.pone.0209191 (PMC6296734; doi:10.1371/journal.pone.0209191)
Supplement: S1 Table — (PDF) [file pone.0209191.s005.pdf]

S1 Table. **Strains and plasmids used in this study**

Strains	Genotype/ phenotype	Reference/ source
E. coli		
DH5α	*recA1 endA1 hsdR17 supE44 thi-1 gyrA96 relA1*D (*lacZYA-argF* U169 [ϕ80d*lacZDM15*] F^- Nal^r)	[1]
NCM631	*hsdS gal* λDE3:*lacI lacUV5:gen1*(T7 RNA polymerase) Δ*lac* linked to Tn10	[2]
P. putida		
KT2440	mt-2 *hsdR*1(r^- m^+)	[3]
IHF3	KT2440 mt-2 *hsdR*1(r^- m^+) *ihfA*::Km	[4]
KT2442	mt-2 *hsdR*1(r^- m^+) Rif^r	[5]
MPO401	mt-2 *hsdR*1 (r^- m^+) Rif^r *cbrB*::Km	[6]
MPO500	mt-2 *hsdR*1 (r^- m^+) Rif^r PP2812::pMO486. Km^r	This work
MPO504	mt-2 *hsdR*1 (r^- m^+) Rif^r PP2812::pMO486. FRT- Km excised	This work

Plasmids	Description	Reference/ source
pET-14b	Expression vector carrying an N-terminal His•Tag® sequence followed by a thrombin cleavage site and three cloning sites under T7 promoter. Ap^r	Novagen
pIZ227	pACYC184 containing lacIq and the T7 lisozyme gene. Cm^r	[2]
pMPO234	Broad-host-range trp–lacZ transcriptional fusion vector, based on pBBR1MCS-4. Ap^r	[7]
pMPO389	Promoter region of *crcY* (coord. positions -273 and +6) into *Sma*I /*BamHI in* pUC18Sfi. Ap^r	This work
pMPO420	Transcriptional fusion of PP2810::lacZ (coord. -472 to -10 from ATG) cloned as EcoRI/BamHI into plasmid pMPO234; Ap^r Cb^r	This work
pMPO422	Transcriptional fusion of PP2810::lacZ (coord. -408 to -10 from ATG) cloned as EcoRI/BamHI into the plasmid pMPO234. Contains a deletion of site F'; Ap^r Cb^r	This work
pMPO425	Substitution of site F1 of *PP2810* promoter (TaTTAa to gcggcg) in plasmid pMPO422. Ap^r Cb^r	This work
pMPO426	Substitution of site R1 of *PP2810* promoter (GTAACA to agcctc) in plasmid pMPO422. Ap^r Cb^r	This work
pMPO428	Substitution of site R2 of *PP2810* promoter (aTAAtg to gcggcg) in plasmid pMPO422. Ap^r Cb^r	This work

pMPO436	Substitution of site F1 of *crcZ* promoter (TGTTAC to gactct) in plasmid pMPO1316. Ap^r Cb^r	This work
pMPO437	Substitution of site R1 of *crcZ* promoter (GTAACA to tgctac) in plasmid pMPO1316. Ap^r Cb^r	This work
pMPO438	Substitution of site R2 of *crcZ* promoter (GTAACg to taccgt) in plasmid pMPO1316. Ap^r Cb^r	This work
pMPO439	Substitution of site F1 of *crcY* promoter (TGTTAC to gactct) in plasmid pMPO1314. Ap^r Cb^r	This work
pMPO440	Substitution of site R1 of *crcY* promoter (acAgCA to tgcgac) in plasmid pMPO1314. Ap^r Cb^r	This work
pMPO441	Substitution of site R' of *crcY* promoter (GTAACA to taccgt) in plasmid pMPO1314. Ap^r Cb^r	This work
pMPO442	Substitution of site R2 of *crcY* promoter (GaAACA to tctcat) in plasmid pMPO1314. Ap^r Cb^r	This work
pMPO486	Coding region of PP2812 (coord. + 123 to +657 from ATG) cloned as BamHI/EcoRI into pMPO1012. Km^r	This work
pMPO1012	Mobilizable expression vector, ColE1, Km^r. Modified from pFPV25 [8]	B. Mesa and A. Flores, unpublished
pMPO1229	Expression vector derived from pET14b based on pT7-7 to overproduce CbrBcloned as NdeI/BamHI.Ap^r	[6]
pMPO1314	Transcriptional fusion of *crcY::lacZ* (coord. -195 and -2 from TSS) in cloned as EcoRI/BamHI into plasmid pMPO234; Ap^r Cb^r	[6]
pMPO1316	Transcriptional fusion of *crcZ::lacZ* (coord. -298 and -1 from TSS) in cloned as EcoRI/BamHI into plasmid pMPO234; Ap^r Cb^r	[6]
pMPO1342	Promoter region of *PP2810* (coord. -394 to -144 from ATG) cloned as a *Sma*I /*Xba*I fragment into pUC18Sfi. Ap^r	This work
pMPO1343	Promoter region of *PP2810* (coord. -334 to -88 from ATG) cloned into *Xba*I/ *Sma*I pUC18Sfi.Ap^r	This work
pMRB117	Promoter sequence of *flgB* (coord. -450 to +128 to ATG) into pMRB3. Ap^r Cb^r	A. Leal and F. Govantes, unpublished
pUC18Sfi	Vector derived from pUC18 with *Sfi*I flanking MCS	[9]

References for supplementary tables

1. Hanahan D. Studies on transformation of Escherichia coli with plasmids. J Mol Biol. 1983;166(4):557-80. Epub 1983/06/05. PubMed PMID: 6345791.
2. Govantes F, Santero E. Transcription termination within the regulatory nifLA operon of Klebsiella pneumoniae. Molecular & general genetics : MGG. 1996;250(4):447-54. Epub 1996/03/07. PubMed PMID: 8602162.
3. Bagdasarian M, Lurz R, Ruckert B, Franklin FC, Bagdasarian MM, Frey J, et al. Specific-purpose plasmid cloning vectors. II. Broad host range, high copy number, RSF1010-derived vectors, and a host-vector system for gene cloning in Pseudomonas. Gene. 1981;16(1-3):237-47. Epub 1981/12/01. PubMed PMID: 6282695.
4. Marques S, Gallegos MT, Manzanera M, Holtel A, Timmis KN, Ramos JL. Activation and repression of transcription at the double tandem divergent promoters for the xylR and xylS genes of the TOL plasmid of Pseudomonas putida. Journal of bacteriology. 1998;180(11):2889-94. Epub 1998/06/06. PubMed PMID: 9603877; PubMed Central PMCID: PMC107254.
5. Franklin FC, Bagdasarian M, Bagdasarian MM, Timmis KN. Molecular and functional analysis of the TOL plasmid pWWO from Pseudomonas putida and cloning of genes for the entire regulated aromatic ring meta cleavage pathway. Proceedings of the National Academy of Sciences of the United States of America. 1981;78(12):7458-62. Epub 1981/12/01. PubMed PMID: 6950388; PubMed Central PMCID: PMC349287.
6. Garcia-Maurino SM, Perez-Martinez I, Amador CI, Canosa I, Santero E. Transcriptional activation of the CrcZ and CrcY regulatory RNAs by the CbrB response regulator in Pseudomonas putida. Molecular microbiology. 2013;89(1):189-205. Epub 2013/05/23. doi: 10.1111/mmi.12270. PubMed PMID: 23692431.
7. Porrua O, Garcia-Gonzalez V, Santero E, Shingler V, Govantes F. Activation and repression of a sigmaN-dependent promoter naturally lacking upstream activation sequences. Molecular microbiology. 2009;73(3):419-33. Epub 2009/07/03. doi: 10.1111/j.1365-2958.2009.06779.x. PubMed PMID: 19570137.
8. Valdivia RH, Falkow S. Bacterial genetics by flow cytometry: rapid isolation of Salmonella typhimurium acid-inducible promoters by differential fluorescence induction. Mol Microbiol. 1996;22(2):367-78. PubMed PMID: 8930920.
9. Herrero M, de Lorenzo V, Timmis KN. Transposon vectors containing non-antibiotic resistance selection markers for cloning and stable chromosomal insertion of foreign genes in gram-negative bacteria. Journal of bacteriology. 1990;172(11):6557-67. Epub 1990/11/01. PubMed PMID: 2172216; PubMed Central PMCID: PMC526845.
