# Supplementary material for: The CbrB Regulon: Promoter dissection reveals novel insights into the CbrAB expression network in Pseudomonas putida
Source: PLoS One. 2018 Dec 17;13(12):e0209191. doi: 10.1371/journal.pone.0209191 (PMC6296734; doi:10.1371/journal.pone.0209191)
Supplement: S2 Table — (PDF) [file pone.0209191.s006.pdf]

S2 Table. **Oligonucleotides used in this study**

Oligonucleotide	Sequence (5' to 3')
crcYBamHI	TTTGGATCCTTGTTGTACCAGAGATA
crcYSmaI	AAACCCGGGCCTGCTGATAGCCGCCG
crcZ49-1Q	AACGGCTCACGCACAATAAAA
crcZ99-2Q	AATCAGTTAGCTGCGCCTCC
crcY72fwd	TTGTGTGGGTGAAGTGTTACCTCCCGCACATTTCACAGCAAA AAATCGGGTAACAGGGAAACAAATCGGCAGAA
crcY72rev	TTTTCTGCCGATTTGTTTCCCTGTTACCCGATTTTTTGCTGTGA AATGTGCGGGAGGTAACACTTCACCCACAC
crcY72mut1fwd	TTGTGTGGGTGAAGGACTCTCTCCCGCACATTTCACAGCAAA AAATCGGGTAACAGGGAAACAAATCGGCAGAA
crcY72mut1rev	TTTTCTGCCGATTTGTTTCCCTGTTACCCGATTTTTTGCTGTGA AATGTGCGGGAGAGAGTCCTTCACCCACAC
crcY72mut2fwd	TTGTGTGGGTGAAGTGTTACCTCCCGCACATTTCTGCGACAA AAATCGGGTAACAGGGAAACAAATCGGCAGAA
crcY72mut2rev	TTTTCTGCCGATTTGTTTCCCTGTTACCCGATTTTTGTCGCAG AAATGTGCGGGAGGTAACACTTCACCCACAC
crcY72mut3fwd	TTGTGTGGGTGAAGTGTTACCTCCCGCACATTTCACAGCAAA AAATCGGTACCGTGGGAAACAAATCGGCAGAA
crcY72mut3rev	TTTTCTGCCGATTTGTTTCCCACGGTACCGATTTTTTGCTGTG AAATGTGCGGGAGGTAACACTTCACCCACAC
crcY72mut4fwd	TTGTGTGGGTGAAGTGTTACCTCCCGCACATTTCACAGCAAA AAATCGGGTAACAGGTCTCATAATCGGCAGAA
crcY72mut4rev	TTTTCTGCCGATTATGAGACCTGTTACCCGATTTTTTGCTGTG AAATGTGCGGGAGGTAACACTTCACCCACAC
crcZ72fwd	TTCCCGTAAAAATCTGTTACCCAAGCTTTCCGCCGTAACAGT CCCCGAGGCGTACGGTAACGAAATTGCGACAT
crcZ72rev	TTATGTCGCAATTTCGTTACCGTACGCCTCGGGGACTGTTAC GGCGGAAAGCTTGGGTAACAGATTTTTACGGG
crcZ72mut1fwd	TTCCCGTAAAAATCGACTCTCCAAGCTTTCCGCCGTAACAGT CCCCGAGGCGTACGGTAACGAAATTGCGACAT
crcZ72mut1rev	TTATGTCGCAATTTCGTTACCGTACGCCTCGGGGACTGTTACG GCGGAAAGCTTGGAGAGTCGATTTTTACGGG
crcZ72mut2fwd	TTCCCGTAAAAATCTGTTACCCAAGCTTTCCGCCTGCTACGT CCCCGAGGCGTACGGTAACGAAATTGCGACAT
crcZ72mut2rev	TTATGTCGCAATTTCGTTACCGTACGCCTCGGGGACGTAGCA GGCGGAAAGCTTGGGTAACAGATTTTTACGG
crcZ72mut3fwd	TTCCCGTAAAAATCTGTTACCCAAGCTTTCCGCCGTAACAG TCCCCGAGGCGTACGTACCGTAAATTGCGACAT
crcZ72mut3rev	TTATGTCGCAATTTACGGTACGTACGCCTCGGGGACTGTTA CGGCGGAAAGCTTGGGTAACAGATTTTTACGGG
EMSA3420HKF	aaatctagaCATCATGGAACGTTTCGGCG
EMSA3420HKR	gggcccgggGAATTCACGCAAGGGTACGG
EMSAoprDF	aaatctagaCGCTTCAGGCGGAAAACAG
EMSAoprDR	tttcccgggTTCCATGTCGCAAATCACGC
foot2810smaF+	TTTcccgggAAAGCCTGCTTTTTTACCGC
foot2810smaR-	TTTcccgggTGATCAACTGACCATGTCAT
foot2810XbaF-	AAAtctagaGTCATGGGCCTGGATAAGC
foot2810XbaR+	TTTtctagaTAGGCGGATTACGCTAGAG
FuscrcZFEco	CCCGAATTCTACCAGCGAATAACCCA
FusioncrcYF	CCGAATTCCAGCGCACAATAGG
FusioncrcYRbisBam	CACGGATCCAGAGATATAGCAGGGT

PP2810EMSAXbaIF	CGGtctagaATGGGTGAATCATTCGCGTC
PP2810EMSASmaR	ACAcccgggAGCGACATGATCAACTGACC
PP2810F	GTTGAATTCGTTGCGGGTTGGTTAACG
PP2810fwmut	TGCGAATTCTCCATGGCGGGCGAAA
PP2810mutIF	GGTGGCGCACGCGGCGAAGGCATCTT
PP2810mutIR	AAGATGCCTTCGCCGCGTGCGCCACC
PP2810mutIIF	GCATCTTTTGAGCCTCCATGTTTCCT
PP2810mutIIIF	CTTACTTCCCGGCGGCGTGCAGGCGC
PP2810mutIIIR	GCGCCTGCACGCCGCCGGGAAGTAAG
PP2810mutIIR	AGGAAACATGGAGGCTCAAAAGATGC
PP2810R	GGAGGATCCGACACTTTCCGACCGT
QflgbF	GGCGCTTGGTATTCACGAAA
QflgBR	ATGTCACGGGCCTTGTAGTT
QPP0952F	TGAAACCATCGCTCGTCCTAA
QPP0952R	TGAGGTGTCATCGTCAGTTGCT
QPP1206F	TCAAGACCCGCATGCTGTATTT
QPP1206R	CGCCTTCGTTGTTACGGAAGT
QPP3009F	TTGCTGTGCTGGTACTCGATCT
QPP3009R	GGATGGCGTTGAGAAACACATA
QPP3074F	ACCCCACAGATCCAGAACATCA
QPP3074R	GGTGGTGTTGAAGAAGGTGCTG
QPP3420F	CCCGTACCCTTGCGTGAAT
QPP3420R	GGCTGATCACCGAAAACCA
QPP3656F	ACACTGCTAGTCGCGCCTTATG
QPP3656R	CCTTTGAAGTCTTCCACGAACG
QPP4050F	CGAACAAGCGCAAGATTCTGT
QPP4050R	TTCACCAGGTCGGCGATTT
QPP4486F	TGTTTTCCCTGGTGTCGCA
QPP4486R	GCTTCGATGCCGATTTTCAG
QPP4643F	CGCTGATCGGCTTTCTGAT
QPP4643R	AACTTGGTGATGCGCGAGA
sec2810cad+	CTAGAGTCATGGGCCTGGATAAGC
sec2810cad-	CTAGATAGGCGGATTACGCTAGAG
Ymut1F	GTGGGTGAAGGACTCTCTCCCGCACA
Ymut1R	TGTGCGGGAGAGAGTCCTTCACCCAC
Ymut2F	CGCACATTTCTGCGACAAAAATCGGG
Ymut2R	CCCGATTTTTGTCGCAGAAATGTGCG
Ymut3F	AAAAAATCGGTACCGTGGGAAACAAA
Ymut3R	TTTGTTTCCCACGGTACCGATTTTTT
Ymut4F	GGGTAACAGGtctcatAATCGGCAGA
Ymut4R	TCTGCCGATTatgagaCCTGTTACCC
ZbisBam	CACGGATCCAAACATATAGCAGGTG
Zmut1F	CGTAAAAATCGACTCTCCAAGCTTTC
Zmut1R	GAAAGCTTGGAGAGTCGATTTTTACG
Zmut2F	GCTTTCCGCCTGCTACGTCCCCGAGG
Zmut2R	CCTCGGGGACGTAGCAGGCGGAAAGC
Zmut3F	GAGGCGTACGTACCGTAAATTGCGAC
Zmut3R	GTCGCAATTTACGGTACGTACGCCTC
